# Interleukin (IL)-25 suppresses IL-22-induced osteoclastogenesis in rheumatoid arthritis via STAT3 and p38 MAPK/IκBα pathway

**DOI:** 10.1186/s13075-020-02315-8

**Published:** 2020-09-23

**Authors:** Hong Ki Min, Ji-Yeon Won, Bo-Mi Kim, Kyung-Ann Lee, Seoung-Joon Lee, Sang-Heon Lee, Hae-Rim Kim, Kyoung-Woon Kim

**Affiliations:** 1 Division of Rheumatology, Department of Internal Medicine, Konkuk University Medical Center, Seoul, 05030 Republic of Korea; 2 R&D Center, OncoInsight, 1022, Gangnam AceTower, 174-10, Jagok-ro, Gangnam-gu, Seoul, 06373 Republic of Korea; 3 Laboratory of Stem Cell, NEXEL, Seoul, Republic of Korea; 4 Division of Rheumatology, Department of Internal Medicine, Soonchunhyang University Hospital, Seoul, 04401 Republic of Korea; 5 Department of Orthopedic Surgery, School of Medicine, Konkuk University, Seoul, 05030 Republic of Korea; 6 Division of Rheumatology, Department of Internal Medicine, Research Institute of Medical Science, School of Medicine, Konkuk University, Seoul, 05030 Republic of Korea

**Keywords:** Rheumatoid arthritis, Osteoclastogenesis, IL-22, IL-25

## Abstract

**Background:**

The present study aimed to evaluate the suppressive role of interleukin (IL)-25 in IL-22-induced osteoclastogenesis and receptor activator of nuclear factor κB ligand (RANKL) expression in rheumatoid arthritis (RA).

**Methods:**

Serum from patients with RA and osteoarthritis (OA), and healthy controls, and synovial fluid from patients with RA and OA were collected, and the levels of IL-22 and IL-25 were measured. RA and OA synovial tissues were stained against IL-25. Fibroblast-like synoviocytes (FLSs) of patients with RA were cultured with IL-22, in the presence or absence of IL-25, and RANKL expression was measured by real-time PCR and enzyme-linked immunosorbent assay (ELISA). Human peripheral blood monocytes were cultured under IL-22/RANKL + M-CSF, with or without IL-25, and tartrate-resistant acid phosphatase (TRAP)-positive cells and osteoclast-related markers were investigated to determine osteoclastogenesis.

**Results:**

Serum and synovial IL-25 levels in RA were upregulated compared to those in OA and healthy control, and elevated expression of IL-25 in RA synovial tissue was re-confirmed. IL-25 and IL-22 levels showed significant correlation in serum and synovial fluid. Pre-treatment of FLS with IL-25 reduced IL-22-induced RANKL expression at the RNA level. The suppressive effects of IL-25 were confirmed to occur through the STAT3 and p38 MAPK/IκBα pathways. IL-25 reduced osteoclast differentiation and suppressed the expression of osteoclast-related markers.

**Conclusion:**

In the current study, we demonstrated the regulatory effect of IL-25 on IL-22-induced osteoclastogenesis. Therapeutic approach involving augmentation of IL-25 regulatory response may serve as a novel treatment option for RA, especially by suppressing osteoclastogenesis.

## Background

Rheumatoid arthritis (RA) is an autoimmune-mediated systemic arthritis. Treatment of RA aims to achieve the following: attainment of low disease activity or remission at the earliest, cessation of structural damage of the affected joints, and prevention of RA-related comorbidities [1]. Pannus is a typical synovial hyperplasia of RA, which can invade into the adjacent articular structure, cartilage, and subchondral bone, thereby inducing erosive joint destruction and deformities [2]. Prevention of such destructive bone erosion is important, primarily due to their irreversible nature. Activation of osteoclasts and secretion of proteases are the main mechanisms underlying cartilage and bone erosion [2]. Maturation and activation of osteoclasts require interaction between receptor activator of nuclear factor κB (RANK) and RANK ligand (RANKL). RANKL can be produced by various cells, although fibroblast-like synoviocyte (FLS), the main cellular component of pannus, is the major source of RANKL in RA synovium [3]. Suppression of RANKL is one of the treatment goals in RA to reduce osteoclastogenesis and eventually cease the irreversible articular damage in RA.

Many pro-inflammatory cytokines, such as tumor necrosis factor (TNF)-α, interleukin (IL)-1β, IL-6, and IL-17A, induce inflammatory processes and bone destruction in RA pathogenesis [4]. Patients with RA, who do not respond to or tolerate conventional synthetic disease-modifying antirheumatic drugs (DMARDs), can use biologic DMARDs instead, which target specific pro-inflammatory cytokines or cell surface molecules [4]. These biologic DMARDs have caused a marked improvement of treatment strategy and clinical remission in RA, although some patients still fail to respond to them, and joint destruction continues to progress. IL-22, a member of the IL-10 superfamily, has recently emerged as a pathological cytokine in animal models of RA [5, 6]. IL-22-producing cluster of differentiation (CD) 4^+^ T cell (Th22) population has been found to be elevated in patients with RA compared to that in healthy controls, and it is also correlated with the disease activity score [7]. Furthermore, IL-22 has been reported to promote FLS proliferation and RANKL expression in FLS, and IL-22-pre-treated FLSs can upregulate osteoclastogenesis [8, 9]. These findings propose a potential therapeutic approach in RA by suppressing IL-22.

IL-25, also called IL-17E, is one of the IL-17 superfamily cytokines, composed of six subtypes, IL-17A to IL-17F; they bind to the corresponding receptor, IL-17 receptor, which in turn is composed of five members, IL-17RA to IL-17RE [10]. Although IL-17 family cytokines share approximately 50% of the amino acid sequence, their cellular responses vary. IL-17A, IL-17C, and IL-17F usually trigger host defense response and promote autoimmune inflammatory response, whereas IL-25 (IL-17E) induces Th2 polarization with allergic response [10]. In recent studies, IL-25 has been shown to present anti-inflammatory response in RA by reducing Th17 differentiation and IL-17-mediated inflammation [11, 12]. The aforementioned findings support an anti-inflammatory role of IL-25 in RA.

In this study, we investigated the expression levels of IL-22 and IL-25 in patients with RA and studied the role of IL-25 in IL-22-induced osteoclastogenesis. Furthermore, the underlying intracellular mechanisms of IL-25 with respect to osteoclastogenesis were evaluated in RA synoviocytes.

## Methods

### Patients

Samples of synovial tissue were isolated from 5 patients with RA (mean age 55.2 ± 3.8 years; range 44–64 years) and 5 with osteoarthritis (OA) patients (mean age 57.8 ± 3.0 years; range 50–68 years), who were undergoing total knee replacement surgery. Synovial fluid was obtained from patients with RA (*N* = 29), who fulfilled the revised criteria of the American College of Rheumatology, 1987 (formerly the American Rheumatism Association), and from patients who had symptomatic knee OA (*N* = 29). Additionally, a total 25 serum of healthy control were included. Informed consent was obtained from all patients, and the experimental protocol was approved by the Konkuk University School of Medicine Human Research Ethics Committee (KUH1010186).

### Isolation of FLS

FLSs were isolated by enzymatic digestion of synovial tissues obtained from patients with RA, who were undergoing total knee replacement surgery, as described previously [13].

### Reagents

IL-22, IL-25, RANKL, and macrophage colony-stimulating factor (M-CSF) were obtained from R&D Systems (Minneapolis, MN, USA).

### Enzyme-linked immunosorbent assay (ELISA) of IL-22, IL-25, and sRANKL

In brief, a 96-well plate (Nunc, Roskilde, Denmark) was coated with 4 μg/ml monoclonal antibodies against IL-22, IL-25, IL-1β, TNF-α, IL-6, IL-4, IL-13, and sRANKL (R&D Systems, Minneapolis, MN, USA) at 4 °C overnight. After blocking with phosphate-buffered saline/1% bovine serum albumin (BSA)/0.05% Tween 20 for 2 h at room temperature (22–25 °C), the test samples and the standard recombinant IL-22, IL-25, IL-1β, TNF-α, IL-6, IL-4, IL-13, and sRANKL (R&D Systems) were added to the 96-well plate and incubated at room temperature for another 2 h. The plates were washed four times with phosphate-buffered saline/Tween 20, and then incubated with 500 ng/ml biotinylated mouse monoclonal antibodies against IL-22, IL-25, IL-1β, TNF-α, IL-6, IL-4, IL-13, and sRANKL (R&D Systems) for 2 h at room temperature. After washing, streptavidin-alkaline phosphate-horseradish peroxidase conjugate (Sigma, St Louis, MA, USA) was incubated for 2 h, followed by another wash, and incubated with 1 mg/ml *p*-nitrophenyl phosphate (Sigma) dissolved in diethanolamine (Sigma) to develop the color reaction. The reaction was stopped by the addition of 1 M NaOH, and optical density of each well was measured at 405 nm. The lower limit of IL-22, IL-25, IL-1β, TNF-α, IL-6, IL-4, IL-13, and sRANKL detection was 10 pg/ml. Recombinant human IL-22, IL-25, IL-1β, TNF-α, IL-6, IL-4, IL-13, and sRANKL, diluted in culture medium, were used as calibration standards, ranging from 10 to 2000 pg/ml. A standard curve was drawn by plotting optical density against log of the concentration of recombinant cytokines, and the curve was used for determining IL-22, IL-25, IL-1β, TNF-α, IL-6, IL-4, IL-13, and sRANKL concentrations in test samples.

### Immunohistochemistry of RA synovium

Immunohistochemical staining for IL-25 was performed with sections of synovium. Briefly, synovial samples were obtained from patients with RA and OA, fixed with 4% paraformaldehyde solution overnight at 4 °C, dehydrated with alcohol, washed, embedded in paraffin, and sectioned into 7-μm-thick slices. Sections were depleted of endogenous peroxidase activity by adding methanolic H_2_O_2_ and blocked with normal serum for 30 min. After overnight incubation with polyclonal anti-human IL-25 antibody (Santa Cruz Biotechnology, Santa Cruz, CA, USA) at 4 °C, the samples were incubated with a secondary antibody, biotinylated anti-rabbit IgG, for 20 min, and then with streptavidin-peroxidase complex (Vector Laboratories, Peterborough, UK) for 1 h, followed by a 5-min incubation with 3,3′-diaminobenzidine (Dako, Glostrup, Denmark). The sections were counterstained with hematoxylin. Samples were finally photographed using an Olympus (Tokyo, Japan) photomicroscope. The area of IL-25^+^ cell from samples was measured in samples using ImageJ software.

### Expression of RANKL mRNA by real-time polymerase chain reaction (PCR)

FLSs were stimulated with various concentrations of IL-22 (0, 1, 10 ng/ml). They were incubated in the presence or absence of IL-25 (10, 50, 100 ng/ml) for 4 h before the addition of IL-22. After 72 h, mRNA levels were measured using real-time PCR, as reported previously [14].

### Western blot analysis

FLSs and PBMC were incubated with IL-22 in the presence or absence of IL-25. After incubation for 1 h, whole-cell lysates were prepared from approximately 2 × 10^5^ cells, by homogenization in the lysis buffer, and then centrifuged at 14,000 rpm for 15 min. Protein concentration in the supernatant was determined using the Bradford method (Bio-Rad, Hercules, CA, USA). Protein samples were separated by 10% sodium dodecyl sulfate-polyacrylamide electrophoresis (SDS-PAGE) and transferred to a nitrocellulose membrane (Amersham Pharmacia Biotech, Uppsala, Sweden). For western blotting, the membrane was pre-incubated with 0.5% skim milk in 0.1% Tween 20 and Tris-buffered saline (TTBS) at room temperature for 2 h. The primary antibodies to phospho-stat3, stat3, phospho-p38, p38, phospho-IκB-α, and IκB-α (Cell Signaling Technology Inc., Danvers, MA, USA), diluted 1:1000 in 5% BSA–0.1% Tween 20/TBS, were added and incubated overnight at 4 °C. The membrane was washed 4 times with TTBS, followed by the addition of horseradish peroxidase-conjugated secondary antibody and incubation for an hour at room temperature. After TTBS washing, hybridized bands were detected using the ECL detection kit and Hyperfilm-ECL reagents (Amersham Pharmacia).

### Osteoclast formation

PBMCs were collected from healthy blood by density gradient separation, and monocytes (osteoclast precursors: pre-OC) were prepared from them. Human monocytes were seeded in 48-well plates at 5 × 10^4^ cells/well with 1 ml of medium. Monocytes were cultured under α-minimum essential medium, 10% heat-inactivated FBS, and 25 ng/ml of recombinant human M-CSF (rhM-CSF) for 3 weeks. Then, monocytes were pre-treated with IL-25 and for 4 h, following which they were added to each well along with IL-22. RANKL was used as the positive control. On day 21, tartrate-resistant acid phosphatase (TRAP)-positive cells were identified, as described previously [14].

### Statistical analysis

All data are expressed as the mean ± standard error of the mean (SEM). Statistical analysis was performed using one-way analysis of variance and Bonferroni’s multiple comparisons test. Spearman’s correlated test was used to seek correlation between cytokine levels. In all analyses, *P* < 0.05 indicated statistical significance.

## Results

### Synovial and serum levels of IL-25 and correlation with IL-22 in patients with RA

**Fig. 1:**
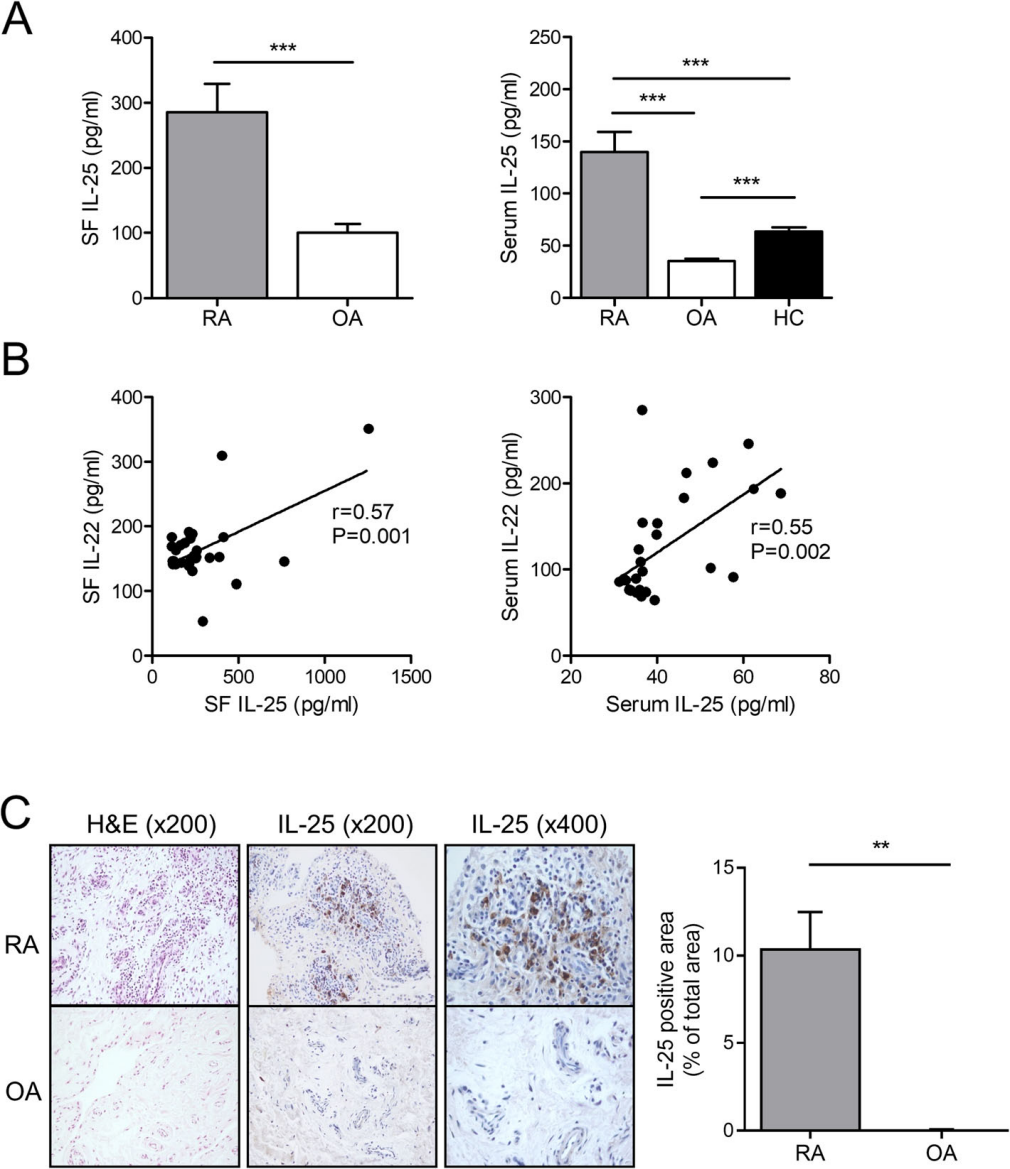
IL-22 and IL-25 expression in serum, synovial fluid, and synovial tissue from patients with rheumatoid arthritis (RA). **a** Concentrations of IL-25 were determined in the synovial fluid and serum of RA and OA, and healthy control subjects. **b** Correlations between the IL-22 and IL-25 concentrations were assessed in the synovial fluid and serum of patients with RA. **c** Expression of IL-25 was detected in the synovium of patients with RA and OA patients using immunohistochemical staining, with hematoxylin and eosin (H&E) counterstaining. Original magnification × 400. ***P < 0.001

Expression of IL-25 in synovial fluid and serum was measured and compared across RA, OA, and healthy control samples (synovial fluid was compared only between patients with RA and OA). Detailed clinical information of RA patients, OA patients, and healthy control was summarized in supplementary Table 1. The level of IL-25 in synovial fluid was significantly higher in RA than in OA cases, and serum IL-25 level was significantly higher in RA than in OA or healthy control samples (Fig. 1a). IL-22 is known to be increased in the serum and synovium of RA cases [7, 8, 15]; we measured the levels of IL-22 and IL-25 in both serum and synovial fluid of patients with RA to reveal whether IL-22 and IL-25 levels are correlated. Serum (*N* = 29) and synovial fluid (*N* = 29) levels of IL-25 showed significant correlation with those of IL-22 (Fig. 1b). Synovial tissue stained against IL-25 presented more number of IL-25-expressing cells in RA than in OA synovium (Fig. 1c). Other cytokines including pro-inflammatory cytokines (TNF-α, IL-1β, and IL-6) and anti-inflammatory cytokines (IL-4 and IL-13) were measured in serum and synovial fluid (supplementary Fig. 1). None of them between IL-25 and TNF-α/IL-1β/IL-6, neither between IL-22 and IL-4/IL-13 showed significant correlation (supplementary Fig. 2 and 3). Furthermore, the levels of IL-1β, TNF-α, IL-6, IL-4, and IL-13 did not change by IL-22 or IL-25 stimulation in RA-FLS (supplementary Fig. 4).

### Suppression of IL-22-mediated RANKL expression in FLS after IL-25 pre-treatment

**Fig. 2:**
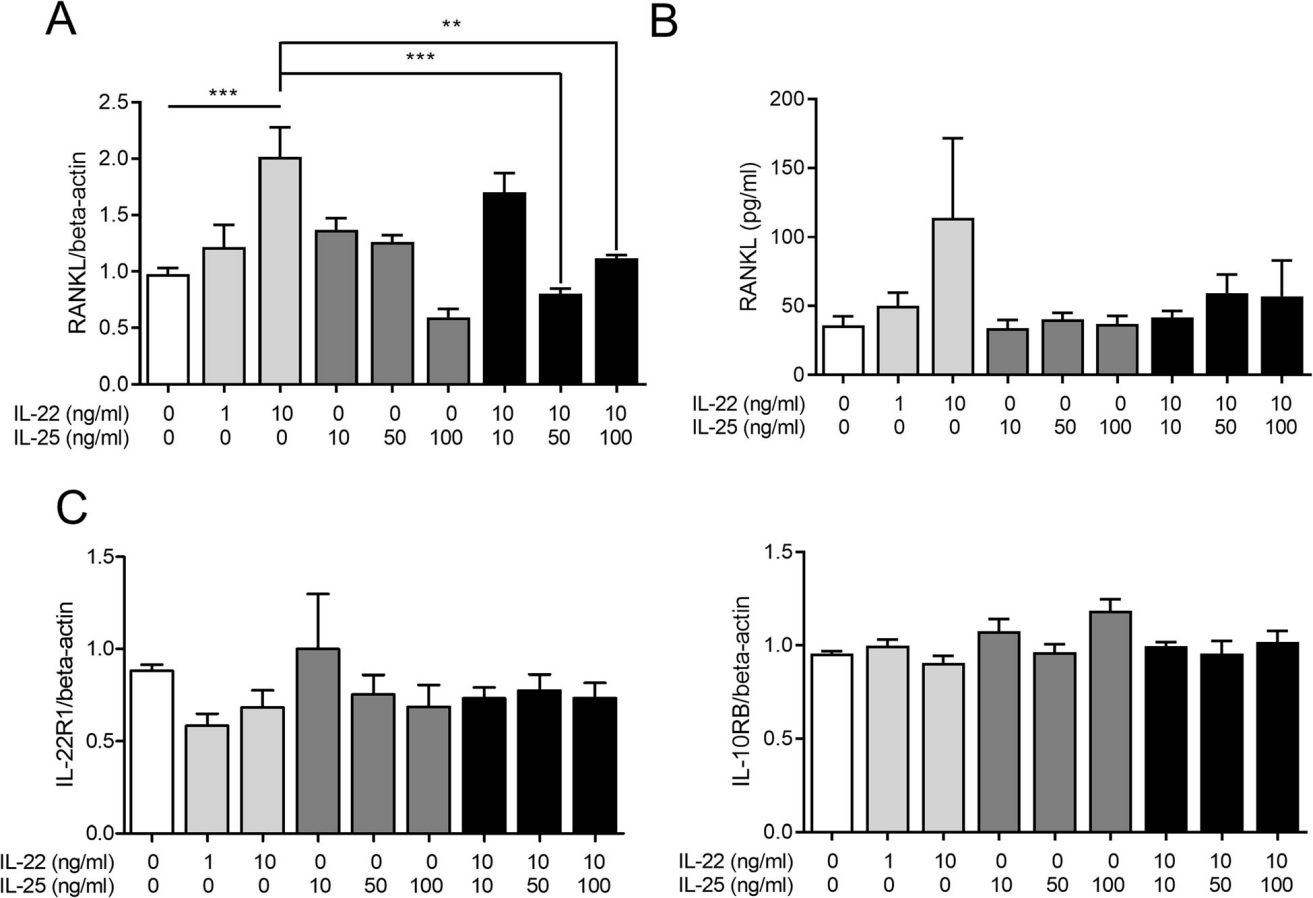
The suppressive effect of IL-25 on RANKL expression in RA synovial fibroblasts. RA synovial fibroblasts were pre-treated with IL-25 and then cultured with 10 ng/ml IL-22 for 72 h. **a** RANKL mRNA level was quantified by real-time PCR. **b** RANKL protein levels were determined by ELISA. **P < 0.01 and ***P < 0.001

Both mRNA and protein levels of RANKL in FLSs were measured by real-time PCR and ELISA, respectively. IL-22 upregulated RANKL mRNA expression in a dose-dependent manner, and pre-treatment with IL-25 significantly suppressed the IL-22-induced overexpression of RANKL (Fig. 2a). The protein levels of RANKL only tended to increase following IL-22 induction and decreased upon IL-25 pre-treatment (Fig. 2b). However, addition of IL-25 did not change mRNA levels of IL-22 receptors, *IL-22R1* and *IL-10RB* (Fig. 2c), which indicates that suppressive role of IL-25 on RANKL expression was independent with expression levels of IL-22 receptor. Co-stimulation of IL-22 and IL-25 (not IL-25 pre-treatment for 4 h) showed similar results (supplementary Fig. 5).

### Intracellular signaling pathway involved in the regulatory function of IL-25 in osteoclastogenesis

**Fig. 3:**
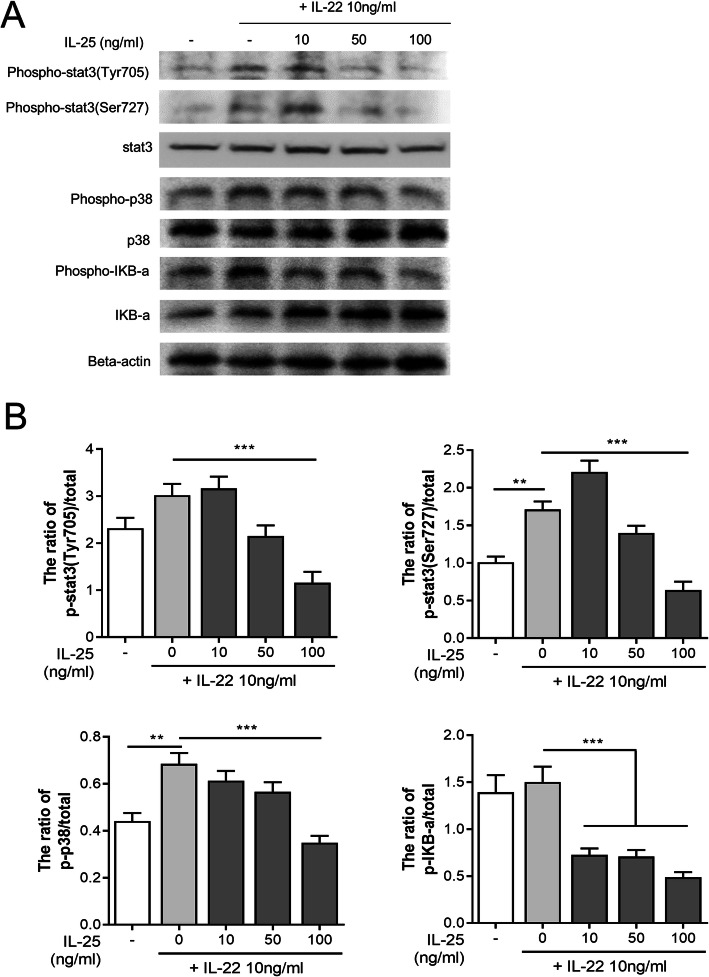
Intracellular signaling pathways involving in the regulation of IL-25 in IL-22-induced RANKL expression in RA synovial fibroblasts. **a** Immunoblotting of *p*-stat3, stat3, *p*-P38, P38, *p-*IκB-α, IκB-α, and beta-actin in the RA synovial fibroblasts pre-treated with IL-25 (10, 50, 100 ng/ml) and then cultured under IL-22 for 1 h. **b** Data were normalized to beta-actin and reported in relative expression units. Bars show the mean ± SEM of 3 independent experiments. *P < 0.05, **P < 0.01, and ***P < 0.001

**Fig. 4:**
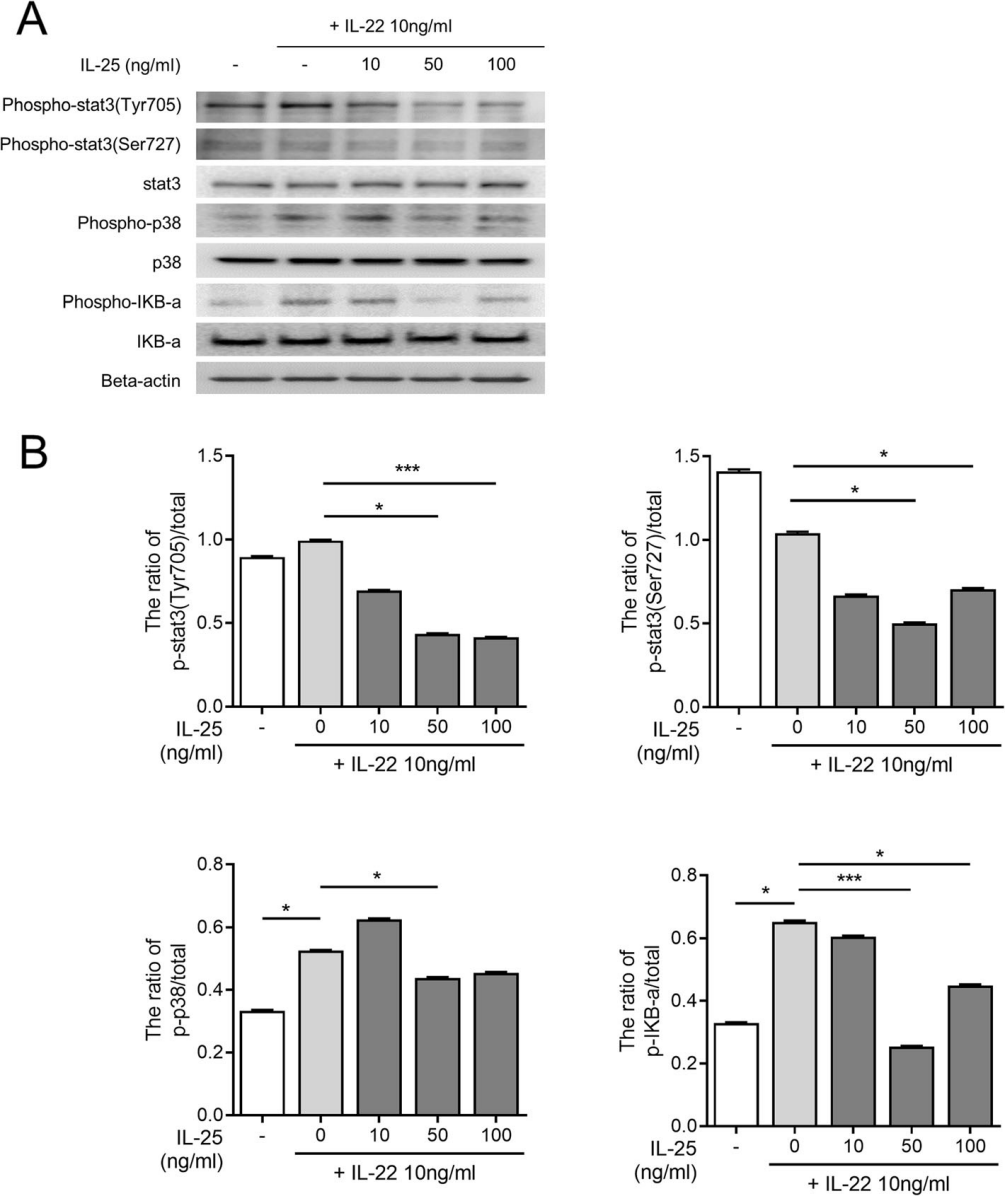
Intracellular signaling pathways involving in the regulation of IL-25 in IL-22-induced RANKL expression in PBMC. **a** Immunoblotting of *p*-stat3, stat3, *p*-P38, P38, *p-*IκB-α, IκB-α, and beta-actin in the PBMC pre-treated with IL-25 (10, 50, 100 ng/ml) and then cultured under IL-22 for 1 h. **b** Data were normalized to beta-actin and reported in relative expression units. Bars show the mean ± SEM of 3 independent experiments. ***P < 0.001

IL-22-induced osteoclastogenesis has been previously shown to be mediated through the p38 MAPK/NF-κB or STAT-3 signaling pathway [8]. IL-22 stimulation alone promoted phosphorylation of STAT3 (Tyr705) and p38, whereas IL-25 pre-treatment downregulated the IL-22-induced phosphorylation of STAT3 (Tyr705, Ser727), p38, and IκB-α in RA-FLS (Fig. 3a). Therefore, the ratio of total to phosphorylated forms of STAT3, p38, and IκB-α significantly decreased (Fig. 3b, raw western blot data in supplementary Fig. 6). The IL-25 stimulation without IL-22 on RA-FLS only suppressed expression level of phosphorylated p38 (supplementary Fig. 7). Pre-treatment of IL-25 with IL-22 stimulation in PBMC suppressed expression of phosphorylated STAT3, p38, and IκB-α (Fig. 4, raw western blot data in supplementary Fig. 8).

### Regulatory effect of IL-25 in osteoclast differentiation from PBMCs

**Fig. 5:**
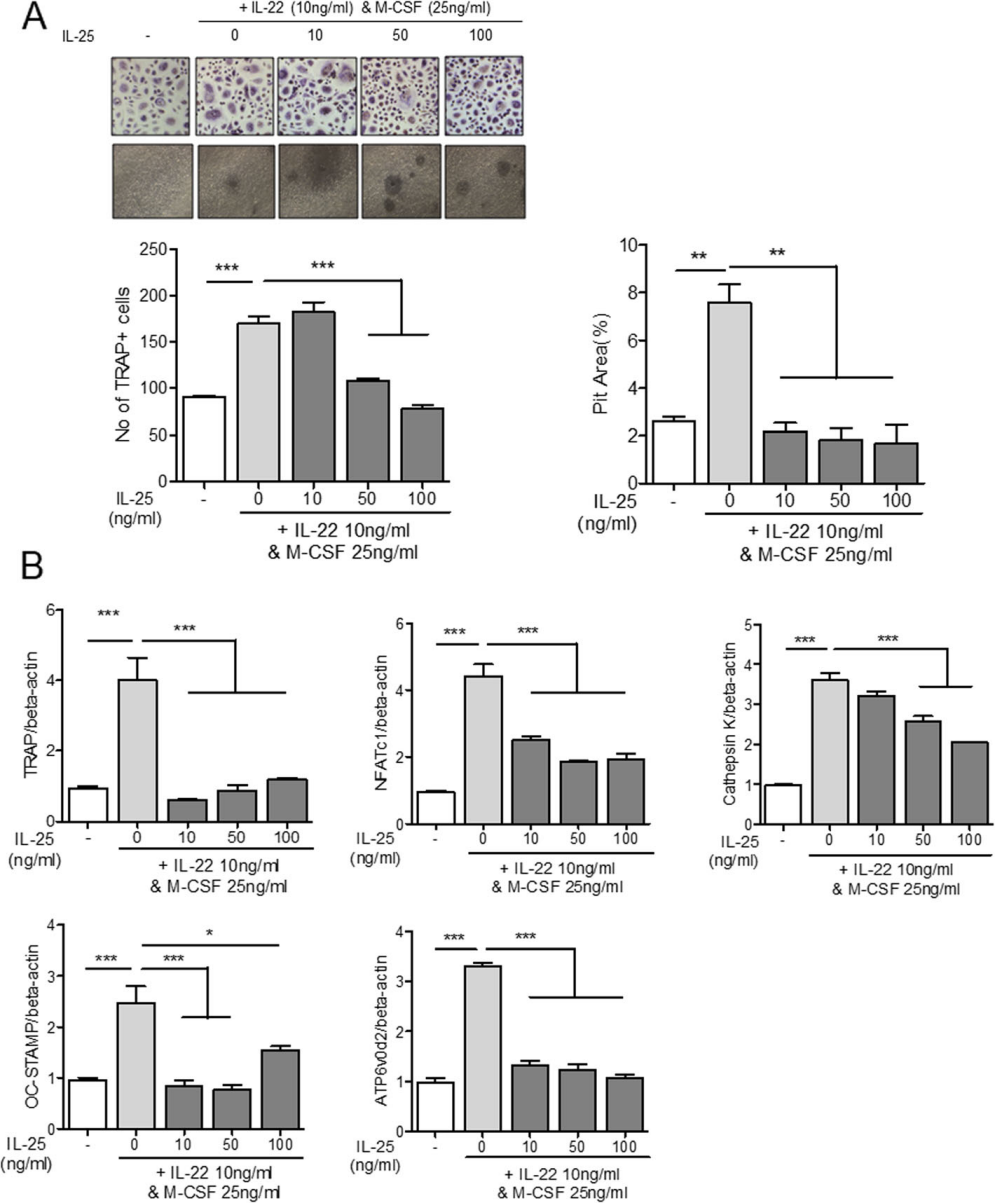
The effect of IL-25 in IL-22-induced osteoclast differentiation from peripheral blood monocytes. CD14^+^ monocytes were pre-treated with IL-25 (0, 10, 50, 100 ng/ml) for 4 h, and then cultured with 25 ng/ml of M-CSF and 10 ng/ml IL-22. **a** TRAP^+^ multinucleated cell count and pit area were measured. **b** The gene expression of TRAP, NFATc1, cathepsin K, OC-STAMP, and ATP6v0d2 from differentiated osteoclasts was measured by real-time PCR. Data were normalized to beta-actin and reported in relative expression units. *P < 0.05, **P < 0.01, and ***P < 0.001

**Fig. 6:**
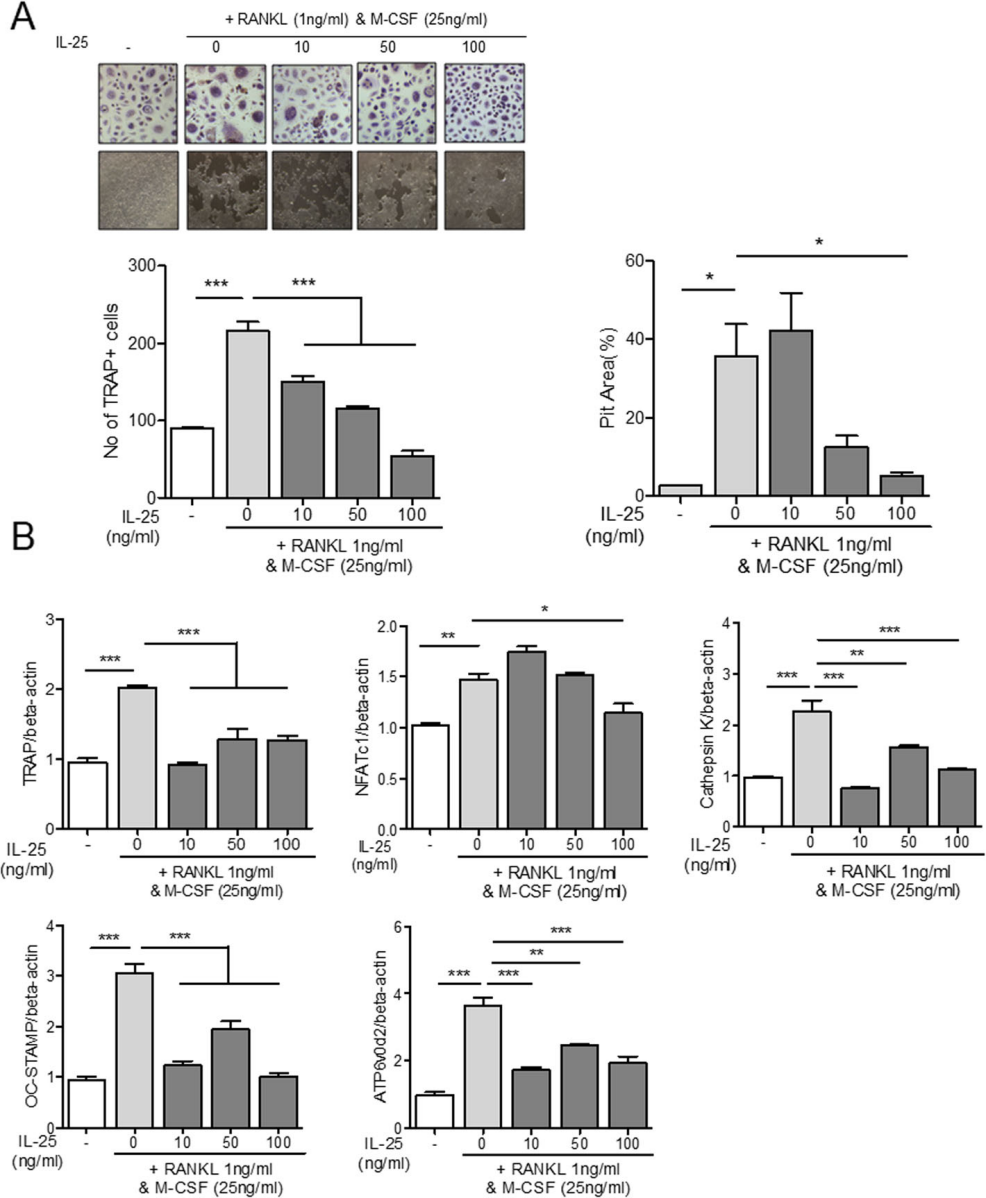
The effect of IL-25 in RANKL-induced osteoclast differentiation from peripheral blood monocytes. CD14^+^ monocytes were pre-treated with IL-25 (0, 10, 50, 100 ng/ml) for 4 h, and then cultured with 25 ng/ml of M-CSF and 1 ng/ml RANKL. **a** TRAP^+^ multinucleated cell count and pit area were measured. **b** The gene expression of TRAP, NFATc1, cathepsin K, OC-STAMP, and ATP6v0d2 from differentiated osteoclasts was measured by real-time PCR. Data were normalized to beta-actin and reported in relative expression units. *P < 0.05, **P < 0.01, and ***P < 0.001

Human PBMCs were cultured under stimulation by IL-22 or RANKL with M-CSF. IL-22 and RANKL effectively induced the differentiation of PBMCs into TRAP^+^ multinucleated osteoclasts (Figs. 5a and 6a). IL-25 suppressed osteoclast differentiation in a dose-dependent manner, in both IL-22- and RANKL-stimulated PBMCs (Figs. 5a and 6a). To investigate the expression of osteoclast-related markers, we evaluated the mRNA levels of TRAP, NFATc1, cathepsin K, OC-STAMP, and ATP6v0d2. Stimulation with either IL-22 or RANKL with M-CSF overexpressed the aforementioned osteoclast-related markers (Figs. 5b and 6b). Co-administration of IL-25, however, lowered the expression levels of all osteoclast markers in IL-22 stimulation (Fig. 5b). Additionally, IL-25 suppressed the osteoclast markers, TRAP, cathepsin K, OC-STAMP, and ATP6v0d2, in RANKL-stimulated conditions (Fig. 6b). NFATc1 expression was significantly suppressed with IL-25-only stimulation at a concentration of 100 ng/ml (Fig. 6b).

## Discussion

IL-22 belongs to the IL-10 family and shares about 25% structural homology with the latter [16]. It can evoke both tissue repair/host defense and inflammatory immune response depending on the organs and diseases [17]. In addition to its diverse functions, it mainly acts on non-hematopoietic cells, such as epithelial cells and fibroblasts, and promotes epithelial cell regeneration. RA synovium has been reported to present high levels of IL-22 expression and is implicated in RA pathogenesis via FLS proliferation and production of monocyte chemoattractant protein 1 (MCP-1) [15]. IL-22 can be expressed by many immune cells, and among the CD4^+^ T cells, Th22 produces over 50% of IL-22 in the peripheral blood [18]. Elevation of IL-22 and Th22 population in patients with RA has been adequately reported, and plasma IL-22 and Th22 levels have shown correlation with RA disease activity (DAS-28) [19–21]. Elevated IL-22 levels in plasma can predict future bone erosion in RA [22], and IL-22 produced by natural killer (NK) cells can induce FLS proliferation [9]. Furthermore, IL-22 has been reported to promote osteoclastogenesis via the p38 MAPK/NF-κB and JAK2/STAT-3 signaling pathways [8]; Th22 cells have been shown to play a crucial role in osteoclastogenesis by producing IL-22 [23]. These findings collectively support the pathological roles of IL-22 in RA pathogenesis and progression. In the current study, besides re-confirming the pathological role of IL-22 in osteoclastogenesis, suppression of IL-22-induced osteoclastogenesis by IL-25 has been revealed for the first time.

IL-25, also called IL-17E, binds to the heterodimeric receptor composed of IL-17RA and IL17RB [10]. It is known to induce Th2 dominant response and cause allergic reaction [24]. Helminth-induced Th2 immune response can suppress inflammatory arthritis and bone loss via the IL-4/IL-13-induced STAT6 pathway [25]. Similarly, IL-25 can attenuate Th17 differentiation in RA in an IL-13-mediated manner [11]. IL-25 has been reported to be produced by synoviocytes in delayed phase after stimulation with IL-17A and TNF-α [12]. Such delayed-phase generation of IL-25 suppresses the production of pro-inflammatory cytokines, IL-6 and IL-17A, in RA synoviocytes [12]. IL-17RB is expressed in various cells, such as NKT, myeloid, Th9, mast, and dendritic cells, as well as basophils, eosinophils, and macrophages [24]. Osteoclast precursor cells, monocytes, have been previously shown to express IL-17RB and IL-17RA in a mouse model [26], as well as in human synoviocytes [12]. In the present study, the novel antagonistic function of IL-25 on osteoclastogenesis induced by IL-22 has been presented.

Elevated plasma and synovial levels of IL-22 and IL-25 in RA have been revealed in previous studies [7, 11, 15, 21, 22]. The regulatory role of IL-25 in RA has been introduced as antagonistic to IL-17A, at a delayed time point when stimulated by TNF-α and IL-17A, in RA [12]. Here, we showed the correlation of IL-22 and IL-25 in the plasma and synovial fluid samples of patients with RA. Considering the regulatory function of IL-25 and the aforementioned correlation with IL-22, IL-25 might be upregulated in response to pathogenic cytokines, such as TNF-α, IL-17A, and IL-22, and antagonize the functions of the pro-inflammatory cytokines. Furthermore, IL-25 is produced by synoviocytes [12], cornerstone component of pannus, and close proximity of synoviocytes with osteoclast precursors makes IL-25 an attractive treatment target.

## Conclusions

In conclusion, the present study showed the suppressive role of IL-25 in osteoclastogenesis in case of RA. Since prevention of bone destruction and bone loss in RA is one of the major treatment targets, upregulation of IL-25 could serve as a novel therapeutic approach for treating RA.

## Supplementary information

**Additional file 1: Supplementary Table 1.** Characteristics of rheumatoid, osteoarthritis patients, and healthy control.

**Additional file 2: Supplementary Figure 1.** Serum and synovial fluid levels of IL-22, IL25, IL-1β, TNF-α, IL-6, IL-4, and IL-13 in RA, OA, and healthy control.

**Additional file 3: Supplementary Figure 2.** Correlation between IL-25 and IL-1β / TNF-α / IL-6 in serum and synovial fluid of RA patients.

**Additional file 4: Supplementary Figure 3.** Correlation between IL-22 and IL-4 / IL-13 in serum and synovial fluid of RA patients.

**Additional file 5: Supplementary Figure 4.** Serum levels of IL-1β, TNF-α, IL-6, IL-4, and IL-13 after stimulation with IL-22 and IL-25 in RA-FLS.

**Additional file 6: Supplementary Figure 5.** The suppressive effect of IL-25 on RANKL expression in RA synovial fibroblasts (IL-22 and IL-25 co-stimulation condition). (A) RANKL mRNA level was quantified by real-time PCR. (B) RANKL protein level were determined by ELISA.

**Additional file 7: Supplementary Figure 6.** Raw western blot data of Fig. 3 (IL-25 pre-treatment with IL-22 stimulation on RA synovial fibroblast).

**Additional file 8: Supplementary Figure 7-1.** Effects of IL-25 stimulation without IL-22 on RA synovial fibroblasts (A) Immunoblotting of *p*-stat3, stat3, *p*-P38, P38, *p-*IκB-α, IκB-α, and beta-actin in the RA synovial fibroblasts with IL-25 single stimulation (10, 50, 100 ng/ml) for 4 hrs. (B) Data were normalized to beta actin and reported in relative expression units. Bars show the mean ± SEM of 3 independent experiments. ^*^P < 0.05, ^**^P < 0.01, and ^***^P < 0.001. **Supplementary Figure 7-2 to 5.** Raw western blot data (IL-25 single stimulation on RA synovial fibroblast).

**Additional file 9: Supplementary Figure 8.** Raw western blot data of Fig. 4 (IL-25 pre-treatment with IL-22 stimulation on PBMC).

## Data Availability

The datasets generated and/or analyzed in this study are available from the corresponding author upon reasonable request.

## Abbreviations

CD: Cluster of differentiation
DMARDs: Disease-modifying antirheumatic drugs
ELISA: Enzyme-linked immunosorbent assay
FLS: Fibroblast-like synoviocyte
IL: Interleukin
OA: Osteoarthritis
PBMC: Peripheral blood mononuclear cell
PCR: Polymerase chain reaction
RA: Rheumatoid arthritis
RANK: Receptor activator of nuclear factor κB
RANKL: Receptor activator of nuclear factor κB ligand
SDS-PAGE: Sodium dodecyl sulfate-polyacrylamide electrophoresis
SEM: Standard error mean
Th: Helper T cell
TNF: Tumor necrosis factor
TRAP: Tartrate-resistant acid phosphatase
TTBS: Tween 20 in Tris-buffered saline
