# Supplementary material for: Interleukin (IL)-25 suppresses IL-22-induced osteoclastogenesis in rheumatoid arthritis via STAT3 and p38 MAPK/IκBα pathway
Source: Arthritis Res Ther. 2020 Sep 23;22:222. doi: 10.1186/s13075-020-02315-8 (PMC7517649; doi:10.1186/s13075-020-02315-8)
Supplement: Supplementary file 1 — Additional file 1: Supplementary Table 1. Characteristics of rheumatoid, osteoarthritis patients, and healthy control. [file 13075_2020_2315_MOESM1_ESM.docx]

Supplementary Table 1. Characteristics of rheumatoid, osteoarthritis patients, and healthy control

|  | RA patients  (N=29) | OA patients  (N=29) | Healthy control (N=25) | *P* |
| --- | --- | --- | --- | --- |
| Age (yeasr) | 58.7 ± 15.8^a^ | 67.2 ± 11.6^b^ | 36.2 ± 11.5^c^ | <0.001^*^ |
| Female, N (%) | 18 (62.1%) | 21 (72.4%) | 18 (72.0%) | 0.636 |
| Menopause, N (%) | 13 (72.2%)^a^ | 17 (81.0%) ^a^ | 3 (15.8%)^b^ | <0.001^**^ |
| Disease duration (years) | 1.9 ± 4.2 | 2.6 ± 2.6 | NA | 0.457 |
| DAS28-ESR | 4.6 ± 1.3 | NA | NA |  |
| DAS28-CRP | 3.5 ± 0.9 | NA | NA |  |
| Rheumatoid factor titer (IU/mL) | 128.1 ± 183.1 | NA | NA |  |
| Anti-CCP (CU) | 528.1 ± 747.4 | NA | NA |  |
| ESR (mm/hr) | 40.0 ± 32.4 | 2.3 ± 3.1 | NA | <0.001† |
| CRP (mg/dL) | 2.2 ± 2.4 | 0.1 ± 0.2 | NA | <0.001† |
| Methotrexate, N (%) | 7 (24.1%) | NA | NA |  |
| Leflunomide, N (%) | 2 ( 6.9%) | NA | NA |  |
| Hydroxychloroquine, N (%) | 3 (10.3%) | NA | NA |  |
| Sulfasalazine, N (%) | 2 ( 6.9%) | NA | NA |  |
| Oral glucocorticoid, N (%) | 7 (24.1%) | NA | NA |  |

* One way analysis of variances were used and post hoc analysis via Bonferroni method. The same letters indicate non-significant difference between groups.

** The Mantel–Haenszel χ2 test was used, and showed significant difference between RA/OA patients, and healthy control.

† Comparison between RA patients and OA patients using Student’s T test.
