# Supplementary figures and images for: Interleukin (IL)-25 suppresses IL-22-induced osteoclastogenesis in rheumatoid arthritis via STAT3 and p38 MAPK/IκBα pathway
Source: Arthritis Res Ther. 2020 Sep 23;22:222. doi: 10.1186/s13075-020-02315-8 (PMC7517649; doi:10.1186/s13075-020-02315-8)

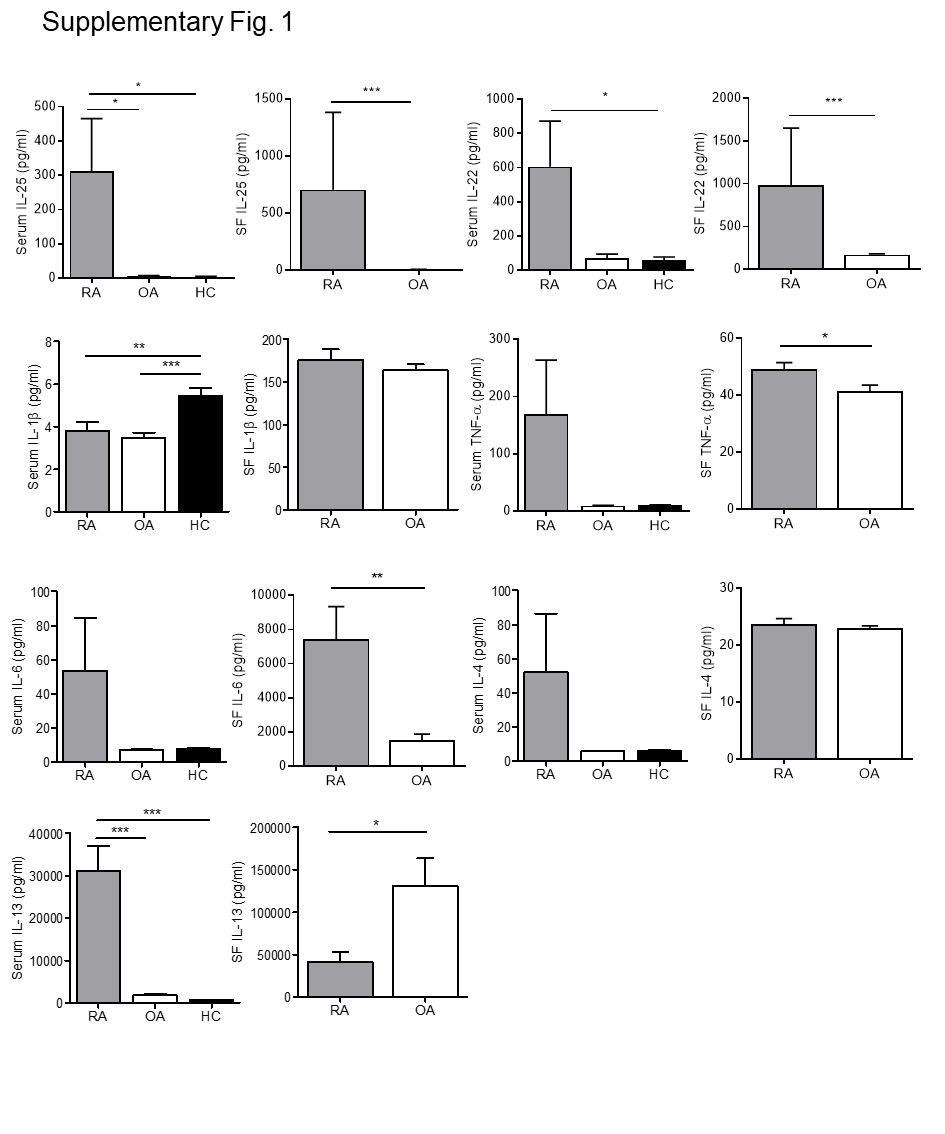

Supplement: Supplementary file 2 — Additional file 2: Supplementary Figure 1. Serum and synovial fluid levels of IL-22, IL25, IL-1β, TNF-α, IL-6, IL-4, and IL-13 in RA, OA, and healthy control. [file 13075_2020_2315_MOESM2_ESM.tif]

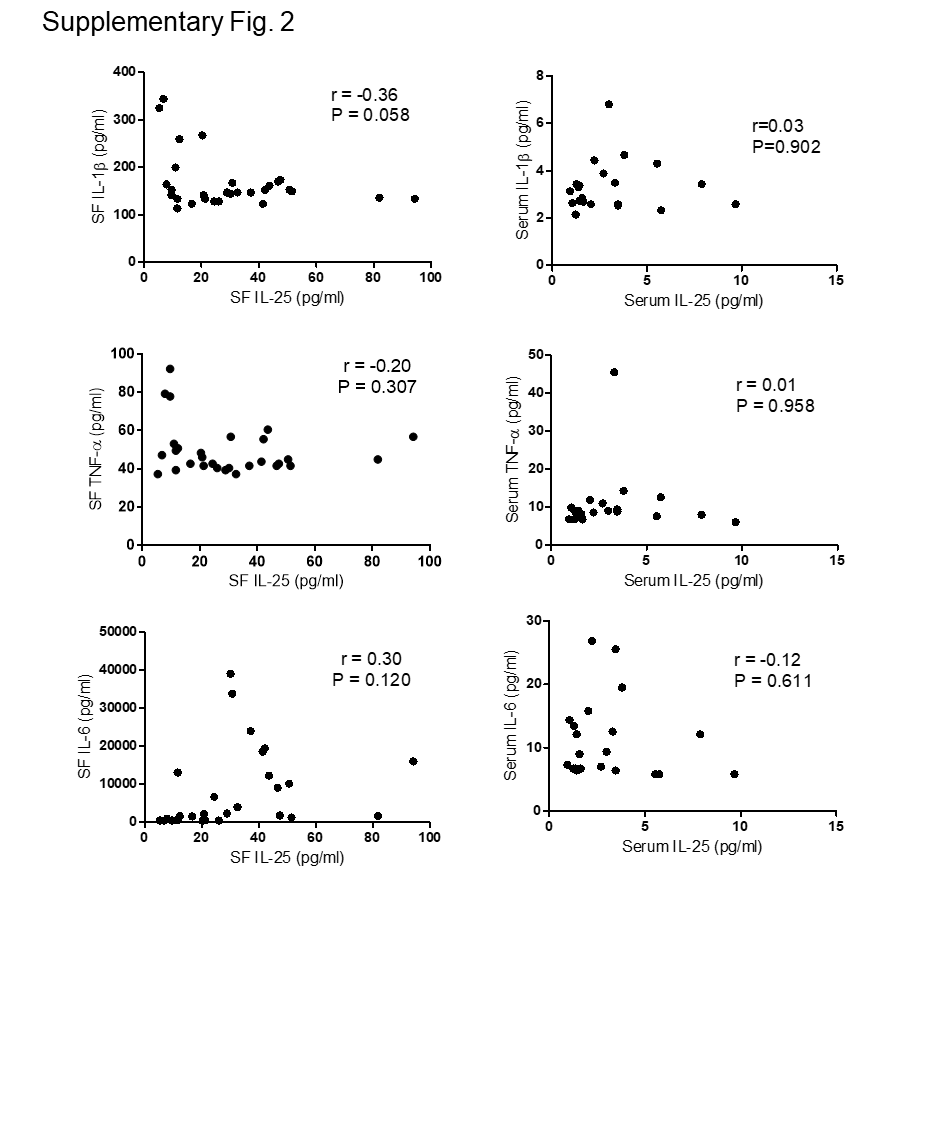

Supplement: Supplementary file 3 — Additional file 3: Supplementary Figure 2. Correlation between IL-25 and IL-1β / TNF-α / IL-6 in serum and synovial fluid of RA patients. [file 13075_2020_2315_MOESM3_ESM.tif]

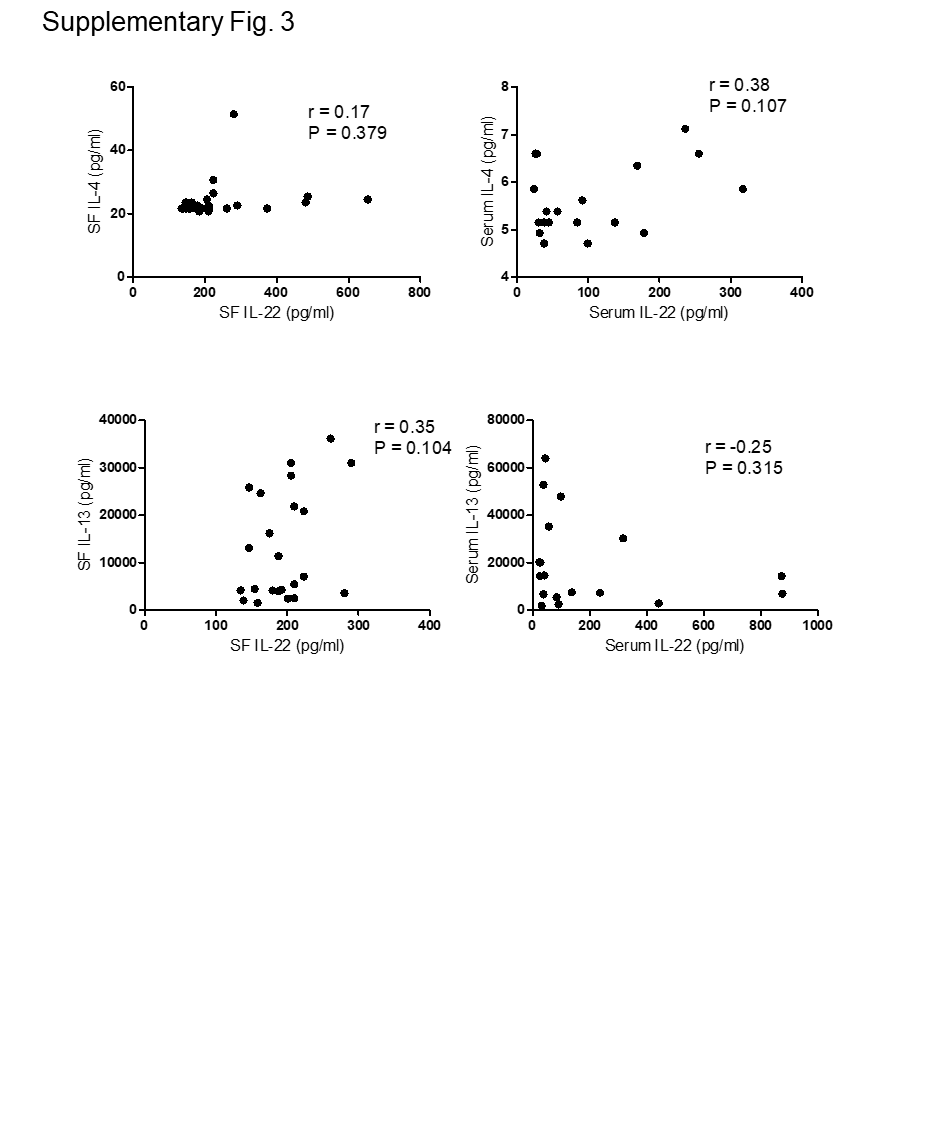

Supplement: Supplementary file 4 — Additional file 4: Supplementary Figure 3. Correlation between IL-22 and IL-4 / IL-13 in serum and synovial fluid of RA patients. [file 13075_2020_2315_MOESM4_ESM.tif]

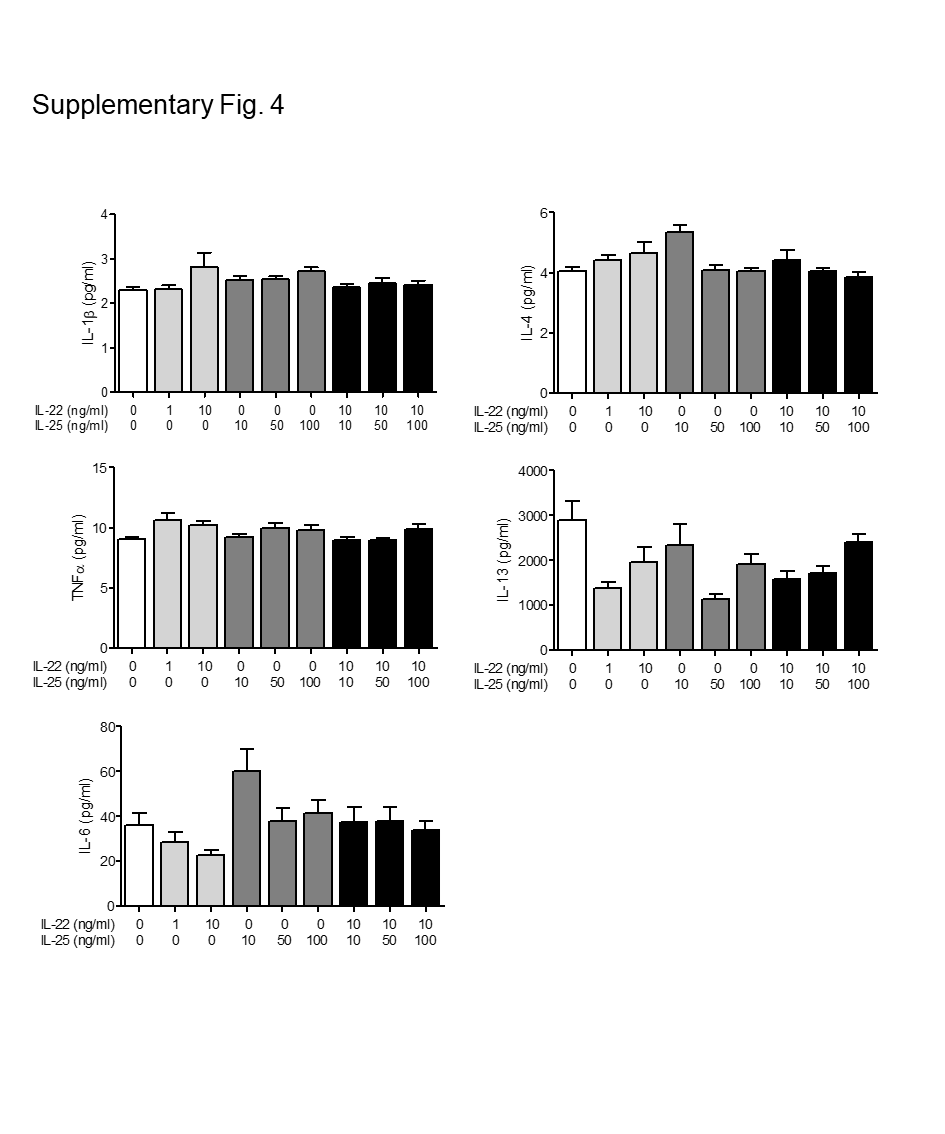

Supplement: Supplementary file 5 — Additional file 5: Supplementary Figure 4. Serum levels of IL-1β, TNF-α, IL-6, IL-4, and IL-13 after stimulation with IL-22 and IL-25 in RA-FLS. [file 13075_2020_2315_MOESM5_ESM.tif]

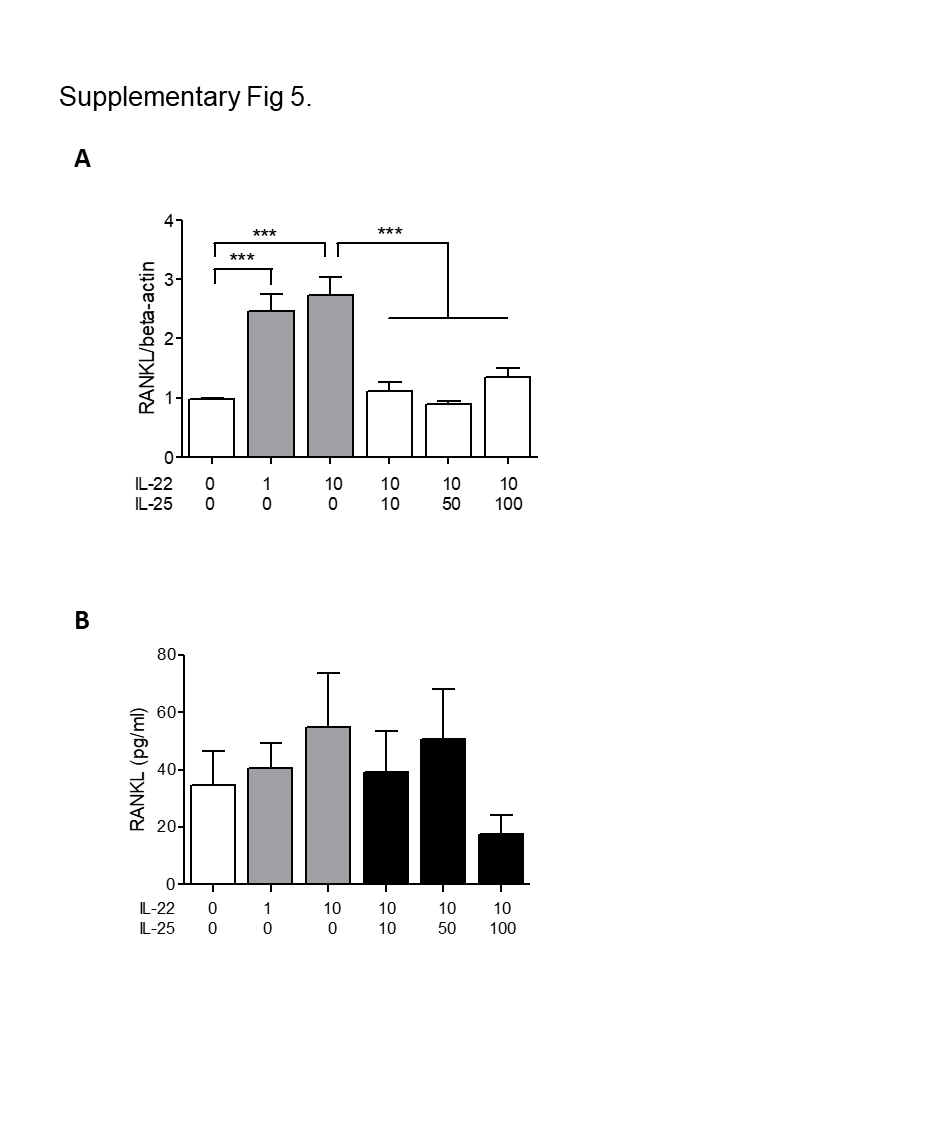

Supplement: Supplementary file 6 — Additional file 6: Supplementary Figure 5. The suppressive effect of IL-25 on RANKL expression in RA synovial fibroblasts (IL-22 and IL-25 co-stimulation condition). (A) RANKL mRNA level was quantified by real-time PCR. (B) RANKL protein level were determined by ELISA. [file 13075_2020_2315_MOESM6_ESM.tif]

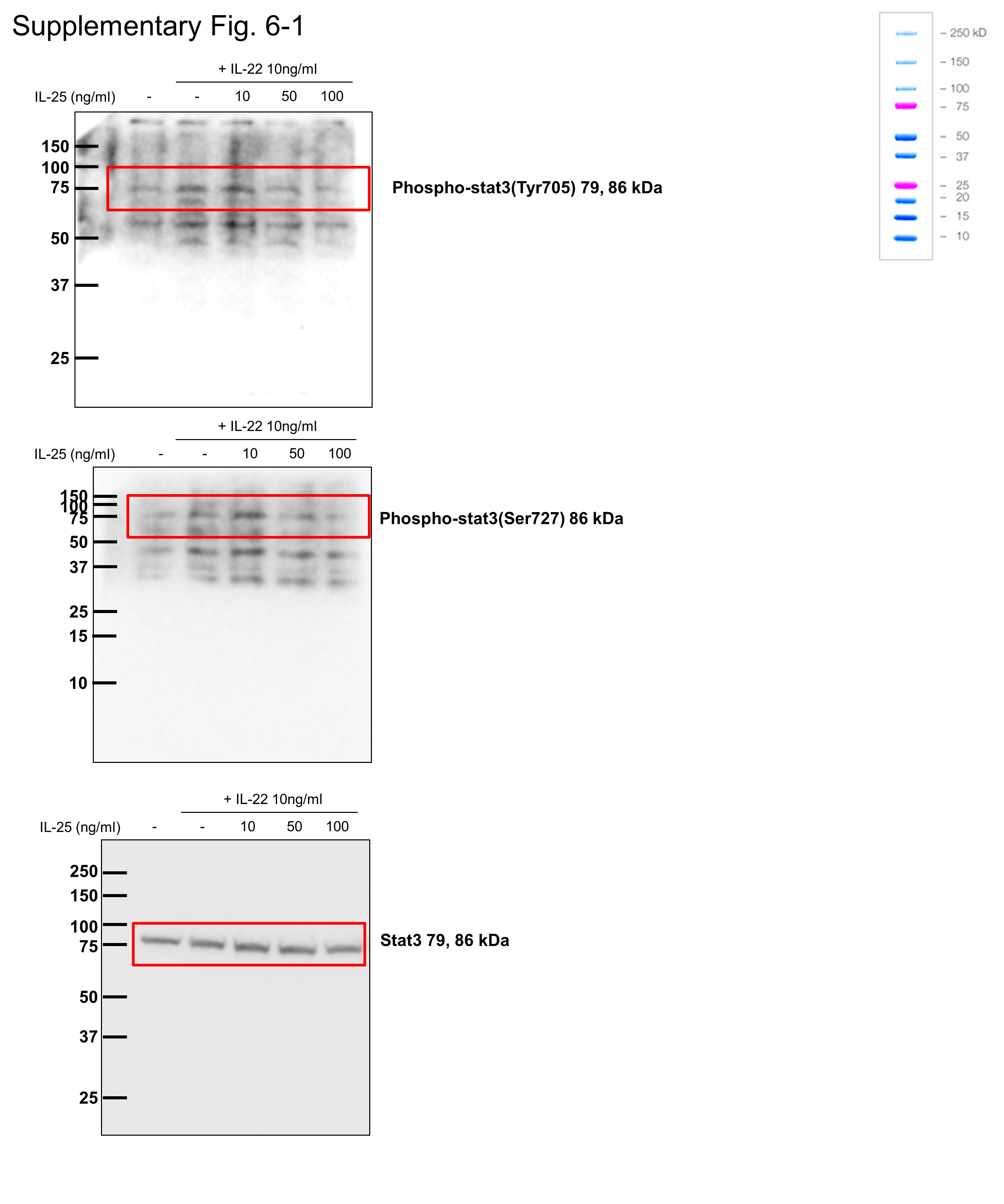

Supplement: Supplementary file 7 — Additional file 7: Supplementary Figure 6. Raw western blot data of Fig. 3 (IL-25 pre-treatment with IL-22 stimulation on RA synovial fibroblast). [file 13075_2020_2315_MOESM7_ESM.zip › Additional file 7-1.tif]

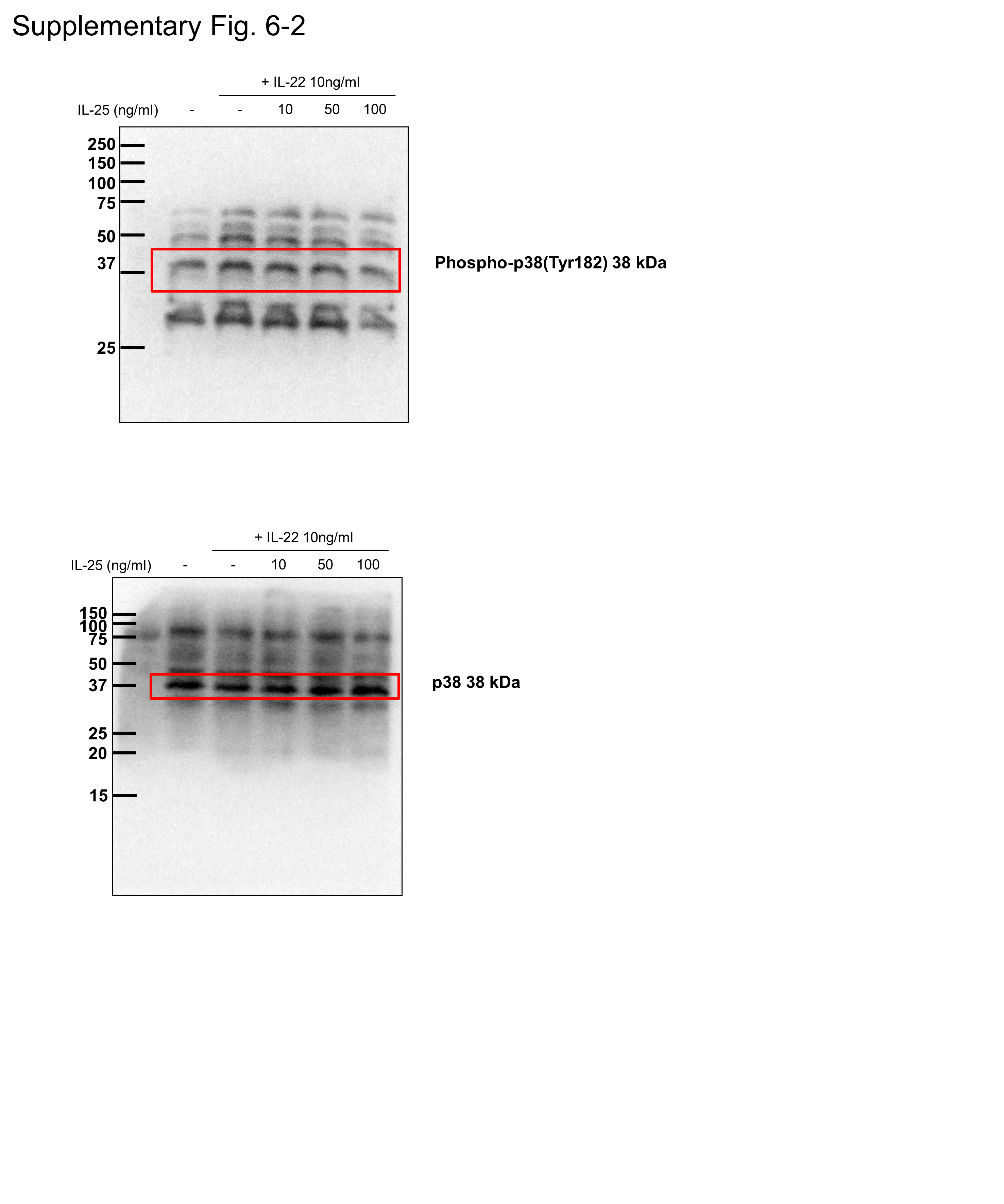

Supplement: Supplementary file 7 — Additional file 7: Supplementary Figure 6. Raw western blot data of Fig. 3 (IL-25 pre-treatment with IL-22 stimulation on RA synovial fibroblast). [file 13075_2020_2315_MOESM7_ESM.zip › Additional file 7-2.tif]

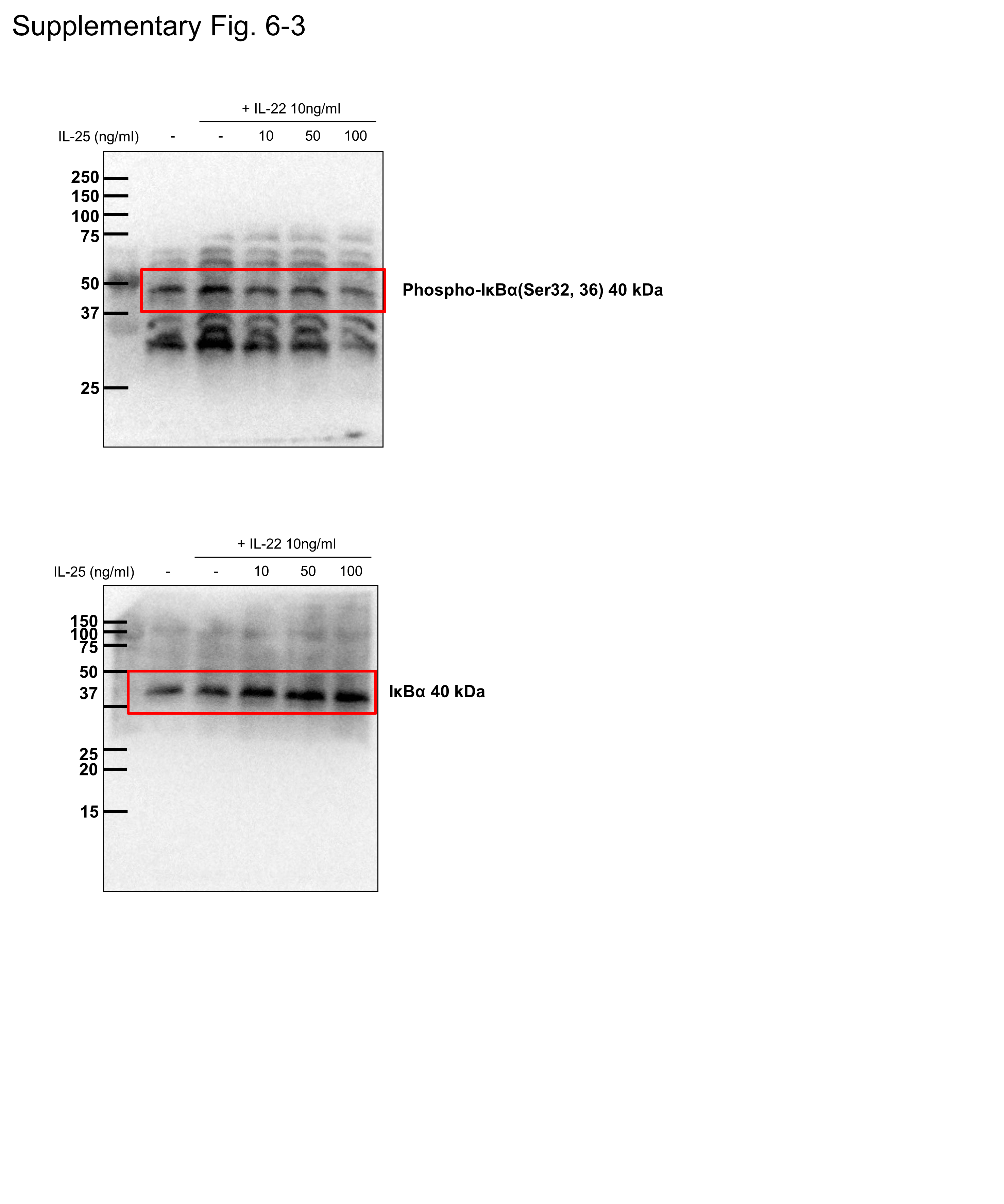

Supplement: Supplementary file 7 — Additional file 7: Supplementary Figure 6. Raw western blot data of Fig. 3 (IL-25 pre-treatment with IL-22 stimulation on RA synovial fibroblast). [file 13075_2020_2315_MOESM7_ESM.zip › Additional file 7-3.tif]

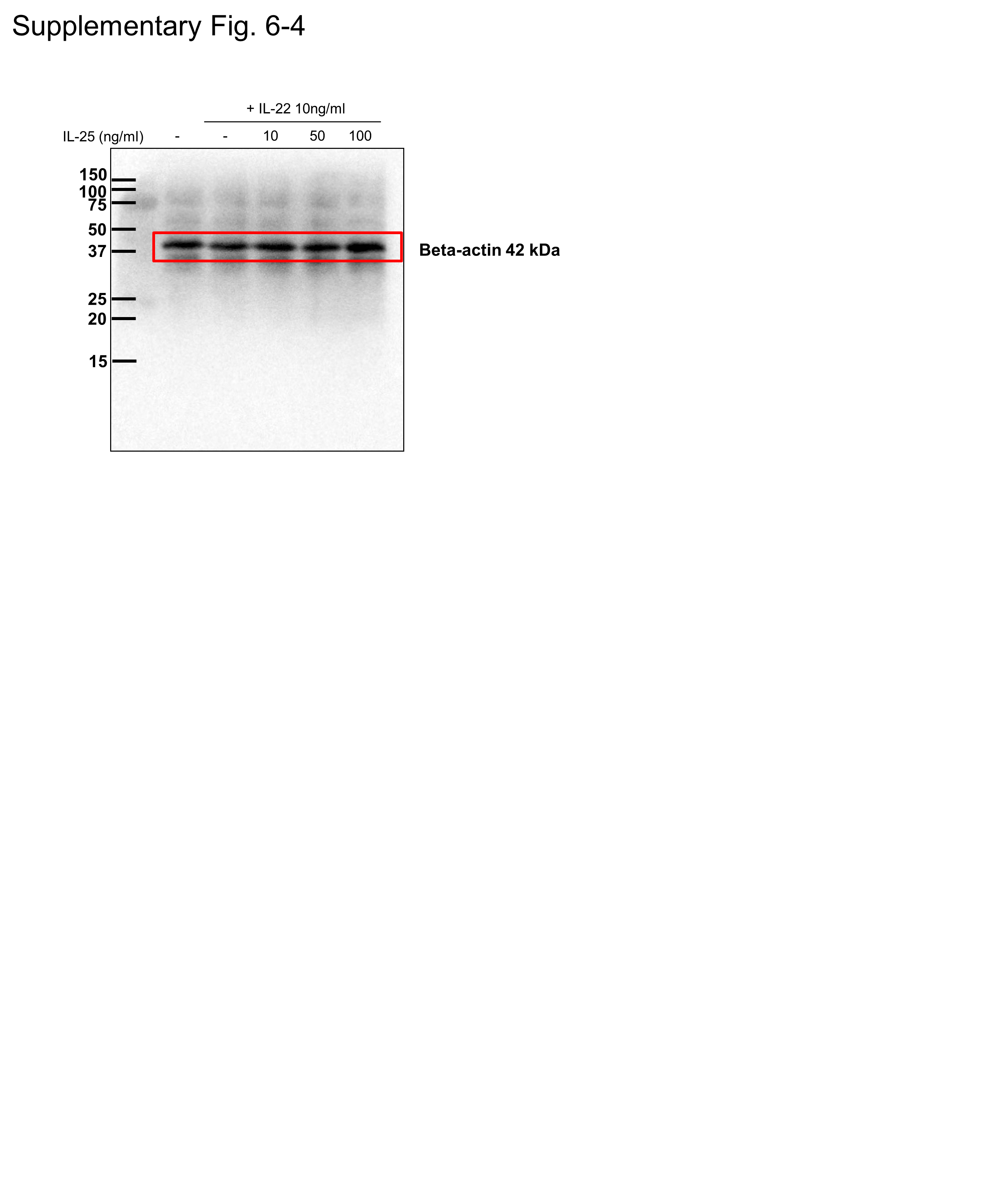

Supplement: Supplementary file 7 — Additional file 7: Supplementary Figure 6. Raw western blot data of Fig. 3 (IL-25 pre-treatment with IL-22 stimulation on RA synovial fibroblast). [file 13075_2020_2315_MOESM7_ESM.zip › Additional file 7-4.tif]

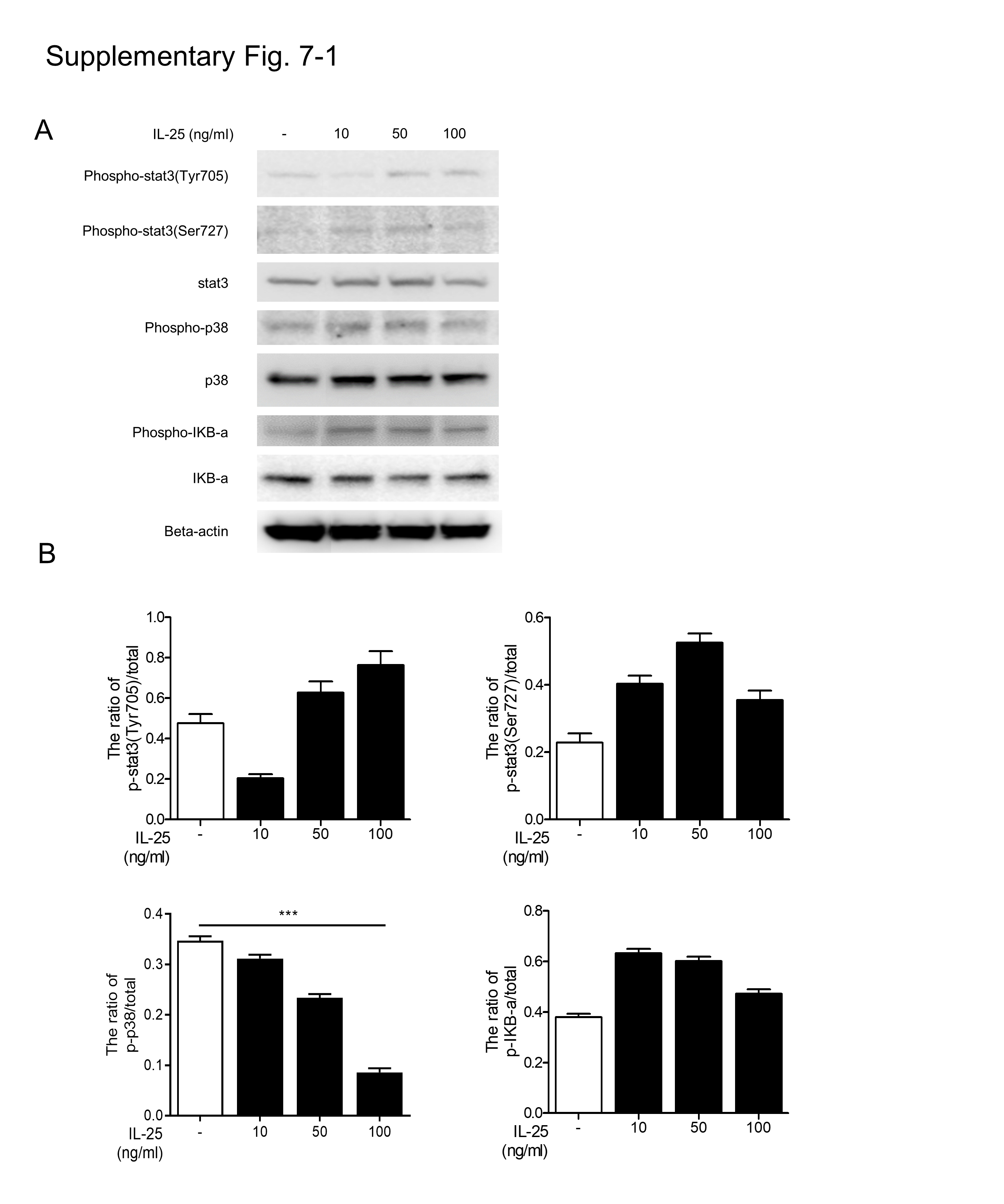

Supplement: Supplementary file 8 — Additional file 8: Supplementary Figure 7-1. Effects of IL-25 stimulation without IL-22 on RA synovial fibroblasts (A) Immunoblotting of p-stat3, stat3, p-P38, P38, p-IκB-α, IκB-α, and beta-actin in the RA synovial fibroblasts with IL-25 single stimulation (10, 50, 100 ng/ml) for 4 hrs. (B) Data were normalized to beta actin and reported in relative expression units. Bars show the mean ± SEM of 3 independent experiments. *P < 0.05, **P < 0.01, and ***P < 0.001. Supplementary Figure 7-2 to 5. Raw western blot data (IL-25 single stimulation on RA synovial fibroblast). [file 13075_2020_2315_MOESM8_ESM.zip › Additional file 8-1.tif]

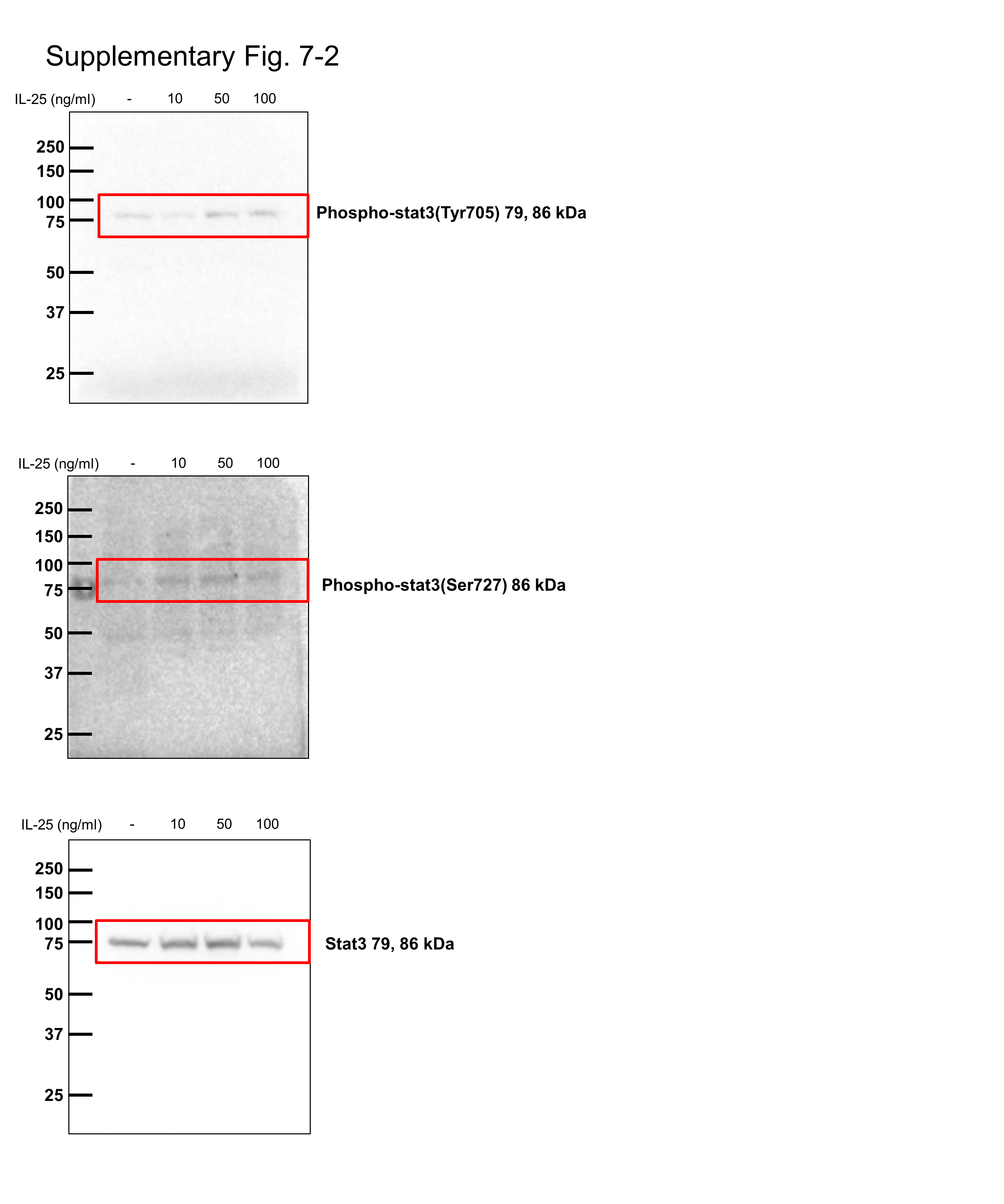

Supplement: Supplementary file 8 — Additional file 8: Supplementary Figure 7-1. Effects of IL-25 stimulation without IL-22 on RA synovial fibroblasts (A) Immunoblotting of p-stat3, stat3, p-P38, P38, p-IκB-α, IκB-α, and beta-actin in the RA synovial fibroblasts with IL-25 single stimulation (10, 50, 100 ng/ml) for 4 hrs. (B) Data were normalized to beta actin and reported in relative expression units. Bars show the mean ± SEM of 3 independent experiments. *P < 0.05, **P < 0.01, and ***P < 0.001. Supplementary Figure 7-2 to 5. Raw western blot data (IL-25 single stimulation on RA synovial fibroblast). [file 13075_2020_2315_MOESM8_ESM.zip › Additional file 8-2.tif]

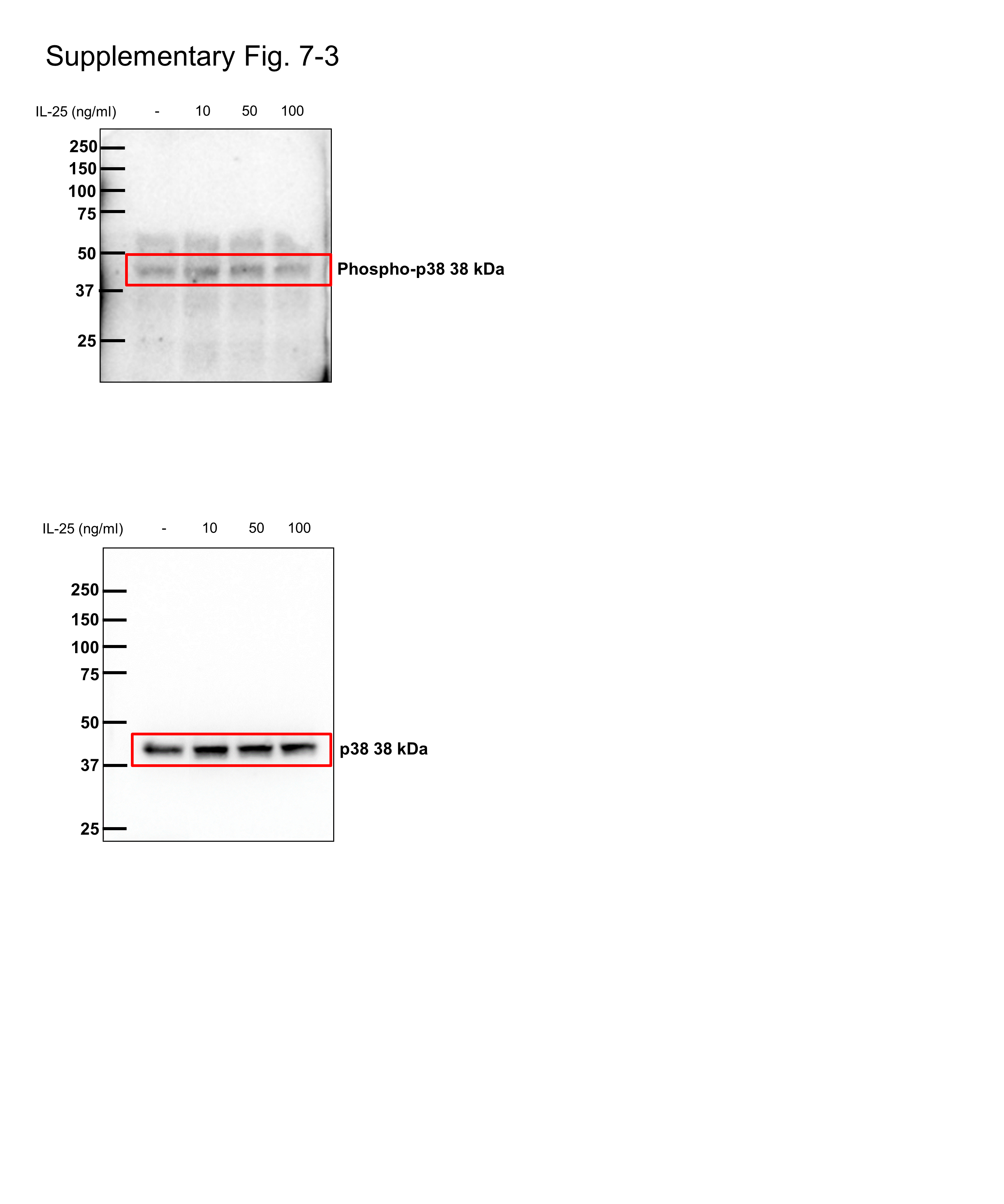

Supplement: Supplementary file 8 — Additional file 8: Supplementary Figure 7-1. Effects of IL-25 stimulation without IL-22 on RA synovial fibroblasts (A) Immunoblotting of p-stat3, stat3, p-P38, P38, p-IκB-α, IκB-α, and beta-actin in the RA synovial fibroblasts with IL-25 single stimulation (10, 50, 100 ng/ml) for 4 hrs. (B) Data were normalized to beta actin and reported in relative expression units. Bars show the mean ± SEM of 3 independent experiments. *P < 0.05, **P < 0.01, and ***P < 0.001. Supplementary Figure 7-2 to 5. Raw western blot data (IL-25 single stimulation on RA synovial fibroblast). [file 13075_2020_2315_MOESM8_ESM.zip › Additional file 8-3.tif]

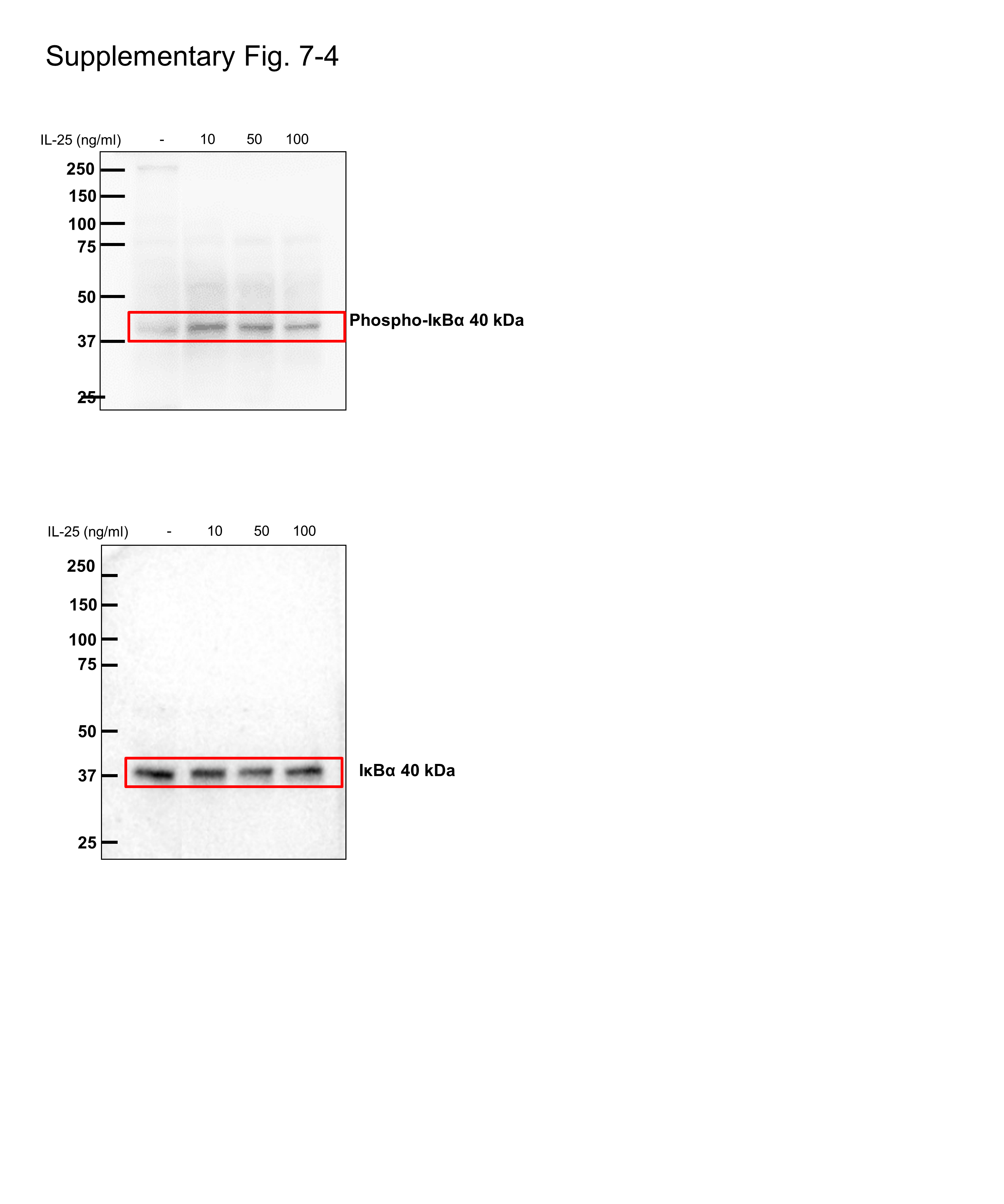

Supplement: Supplementary file 8 — Additional file 8: Supplementary Figure 7-1. Effects of IL-25 stimulation without IL-22 on RA synovial fibroblasts (A) Immunoblotting of p-stat3, stat3, p-P38, P38, p-IκB-α, IκB-α, and beta-actin in the RA synovial fibroblasts with IL-25 single stimulation (10, 50, 100 ng/ml) for 4 hrs. (B) Data were normalized to beta actin and reported in relative expression units. Bars show the mean ± SEM of 3 independent experiments. *P < 0.05, **P < 0.01, and ***P < 0.001. Supplementary Figure 7-2 to 5. Raw western blot data (IL-25 single stimulation on RA synovial fibroblast). [file 13075_2020_2315_MOESM8_ESM.zip › Additional file 8-4.tif]

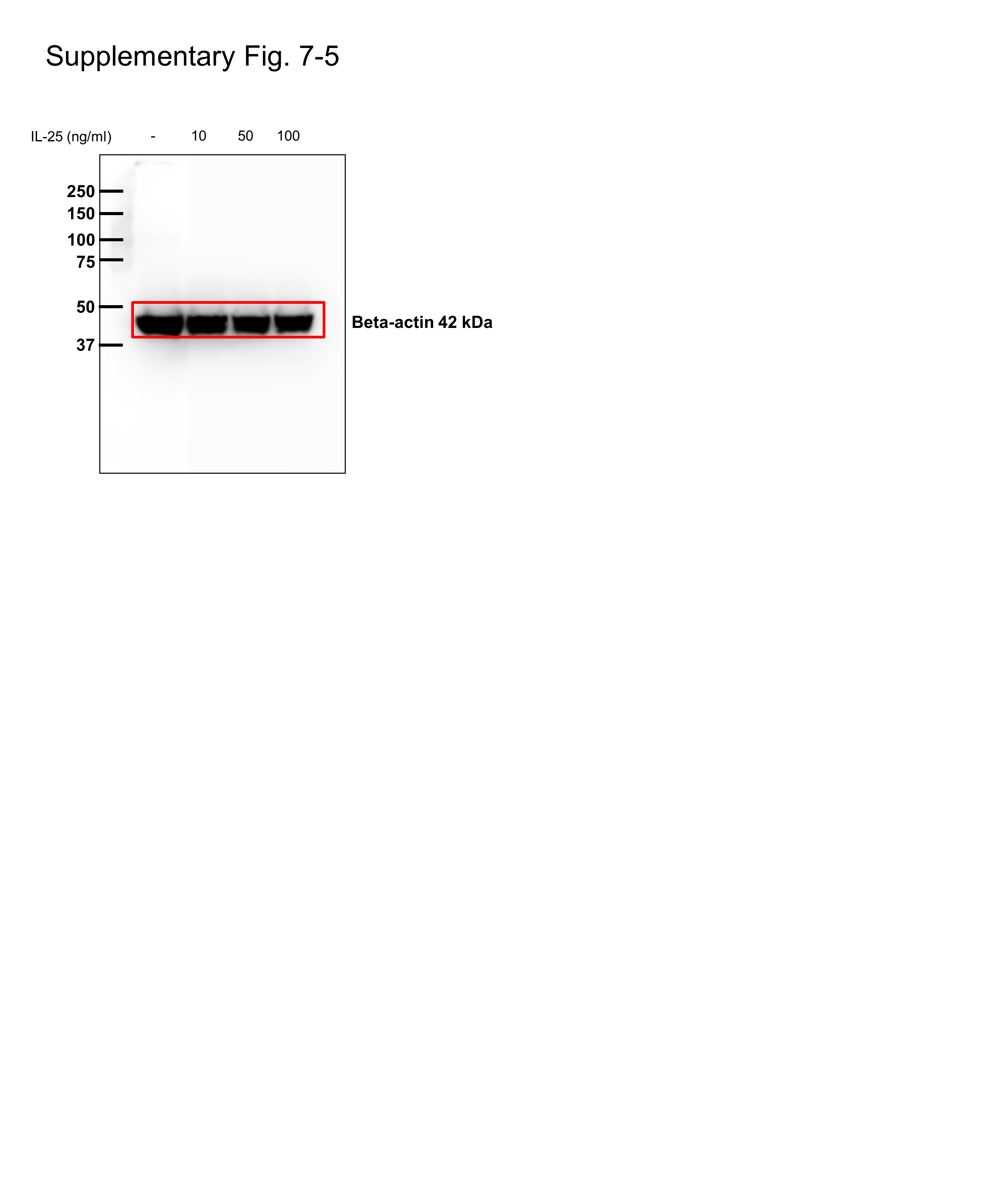

Supplement: Supplementary file 8 — Additional file 8: Supplementary Figure 7-1. Effects of IL-25 stimulation without IL-22 on RA synovial fibroblasts (A) Immunoblotting of p-stat3, stat3, p-P38, P38, p-IκB-α, IκB-α, and beta-actin in the RA synovial fibroblasts with IL-25 single stimulation (10, 50, 100 ng/ml) for 4 hrs. (B) Data were normalized to beta actin and reported in relative expression units. Bars show the mean ± SEM of 3 independent experiments. *P < 0.05, **P < 0.01, and ***P < 0.001. Supplementary Figure 7-2 to 5. Raw western blot data (IL-25 single stimulation on RA synovial fibroblast). [file 13075_2020_2315_MOESM8_ESM.zip › Additional file 8-5.tif]

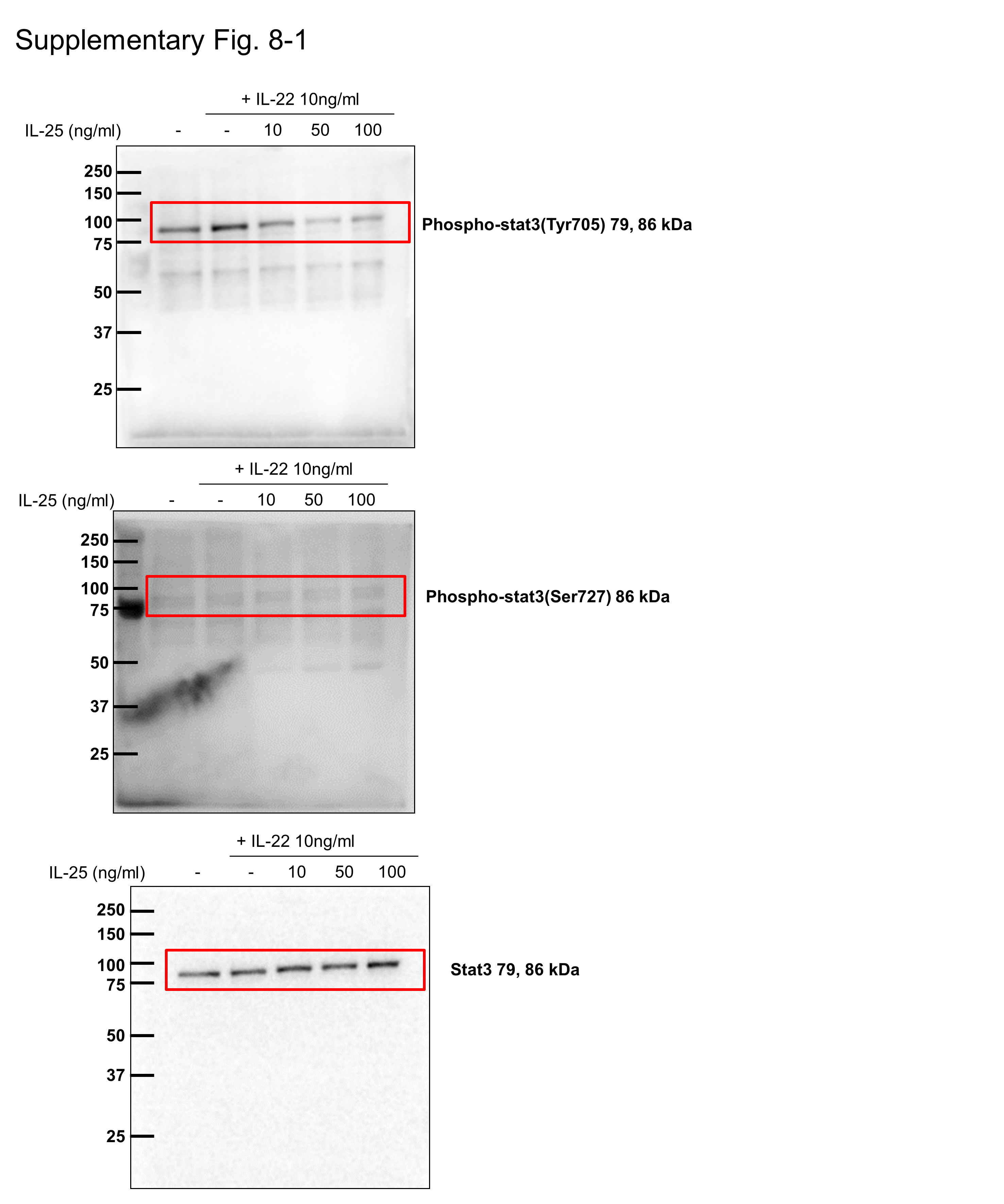

Supplement: Supplementary file 9 — Additional file 9: Supplementary Figure 8. Raw western blot data of Fig. 4 (IL-25 pre-treatment with IL-22 stimulation on PBMC). [file 13075_2020_2315_MOESM9_ESM.zip › Additional file 9-1.tif]

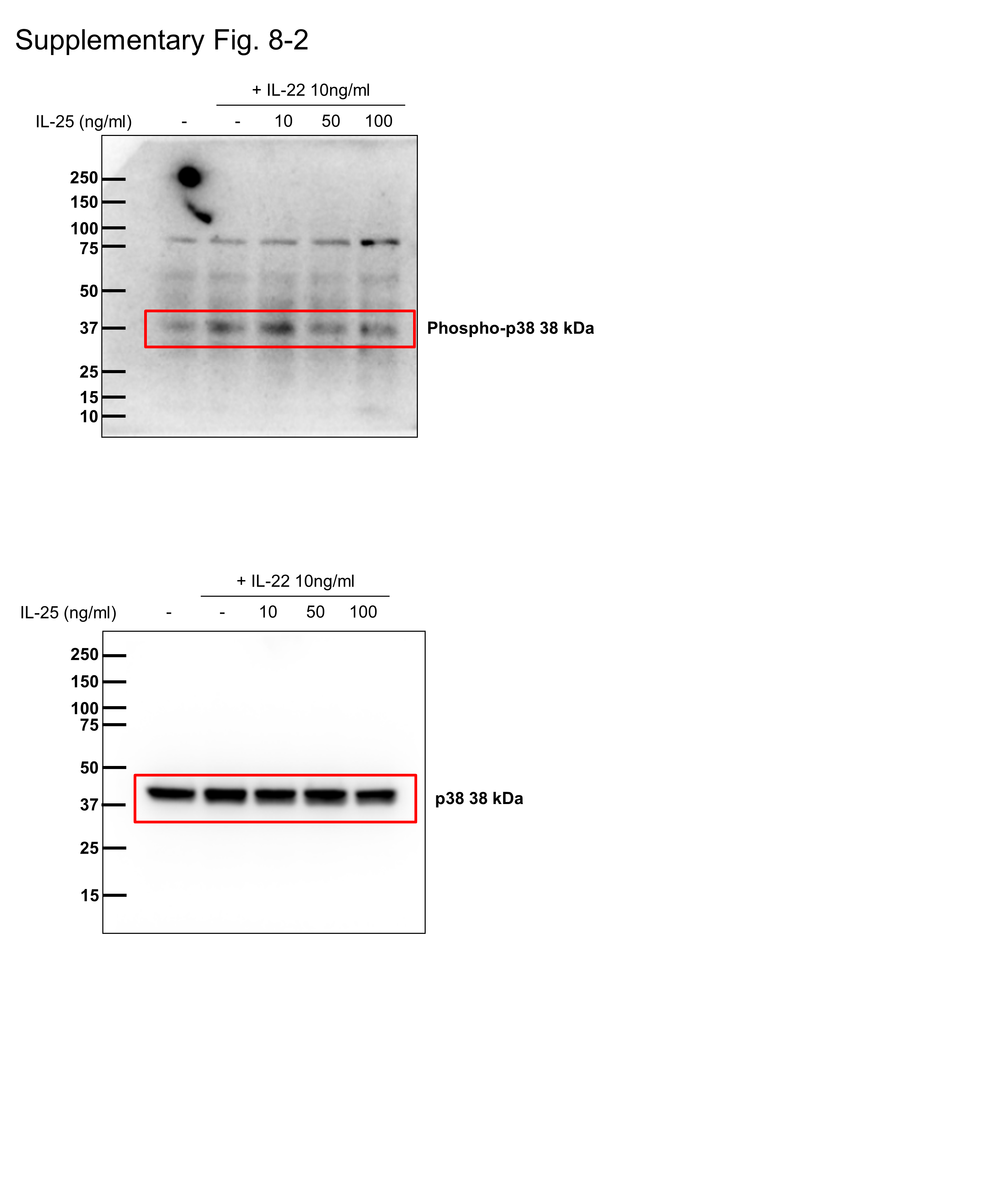

Supplement: Supplementary file 9 — Additional file 9: Supplementary Figure 8. Raw western blot data of Fig. 4 (IL-25 pre-treatment with IL-22 stimulation on PBMC). [file 13075_2020_2315_MOESM9_ESM.zip › Additional file 9-2.tif]

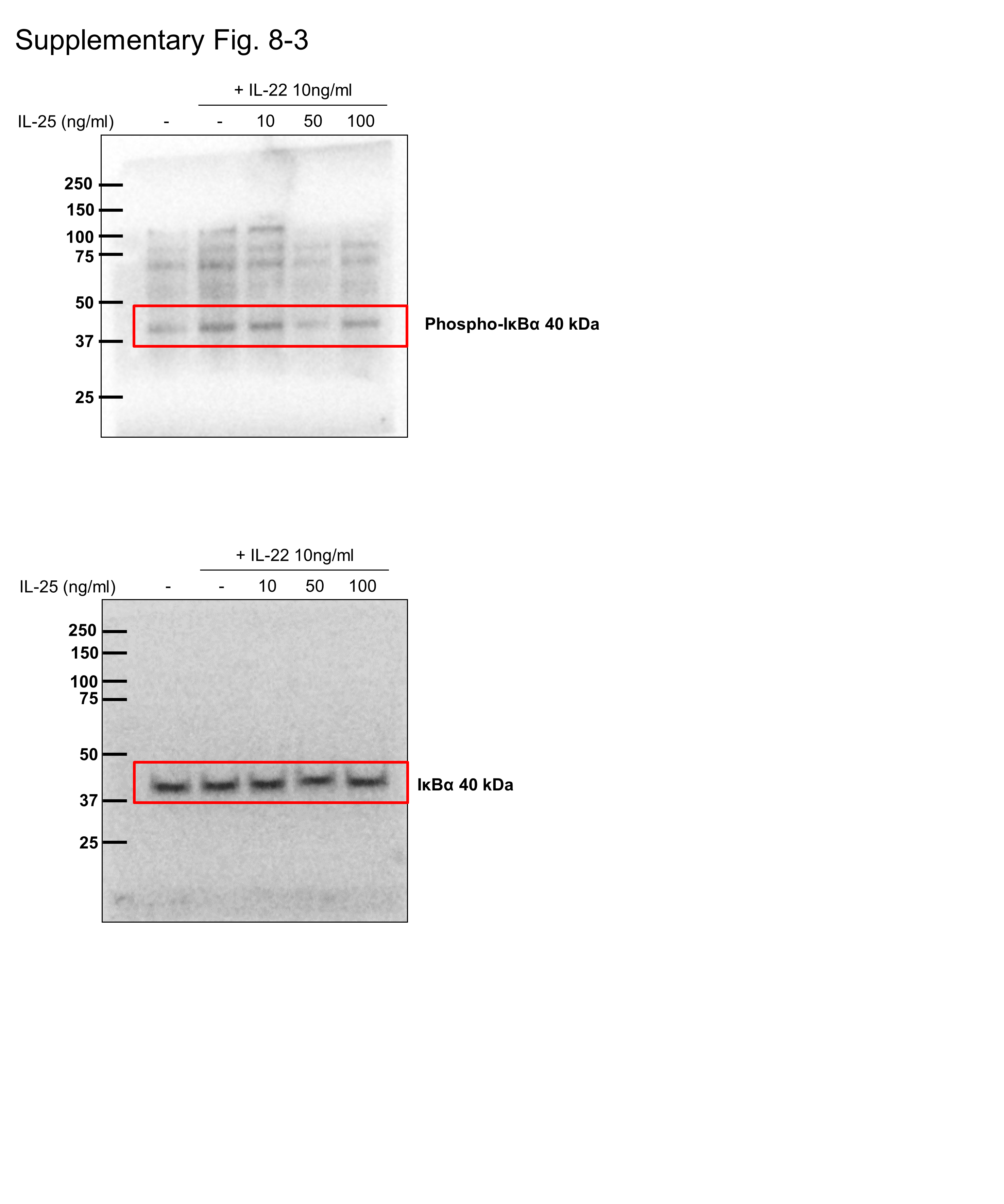

Supplement: Supplementary file 9 — Additional file 9: Supplementary Figure 8. Raw western blot data of Fig. 4 (IL-25 pre-treatment with IL-22 stimulation on PBMC). [file 13075_2020_2315_MOESM9_ESM.zip › Additional file 9-3.tif]

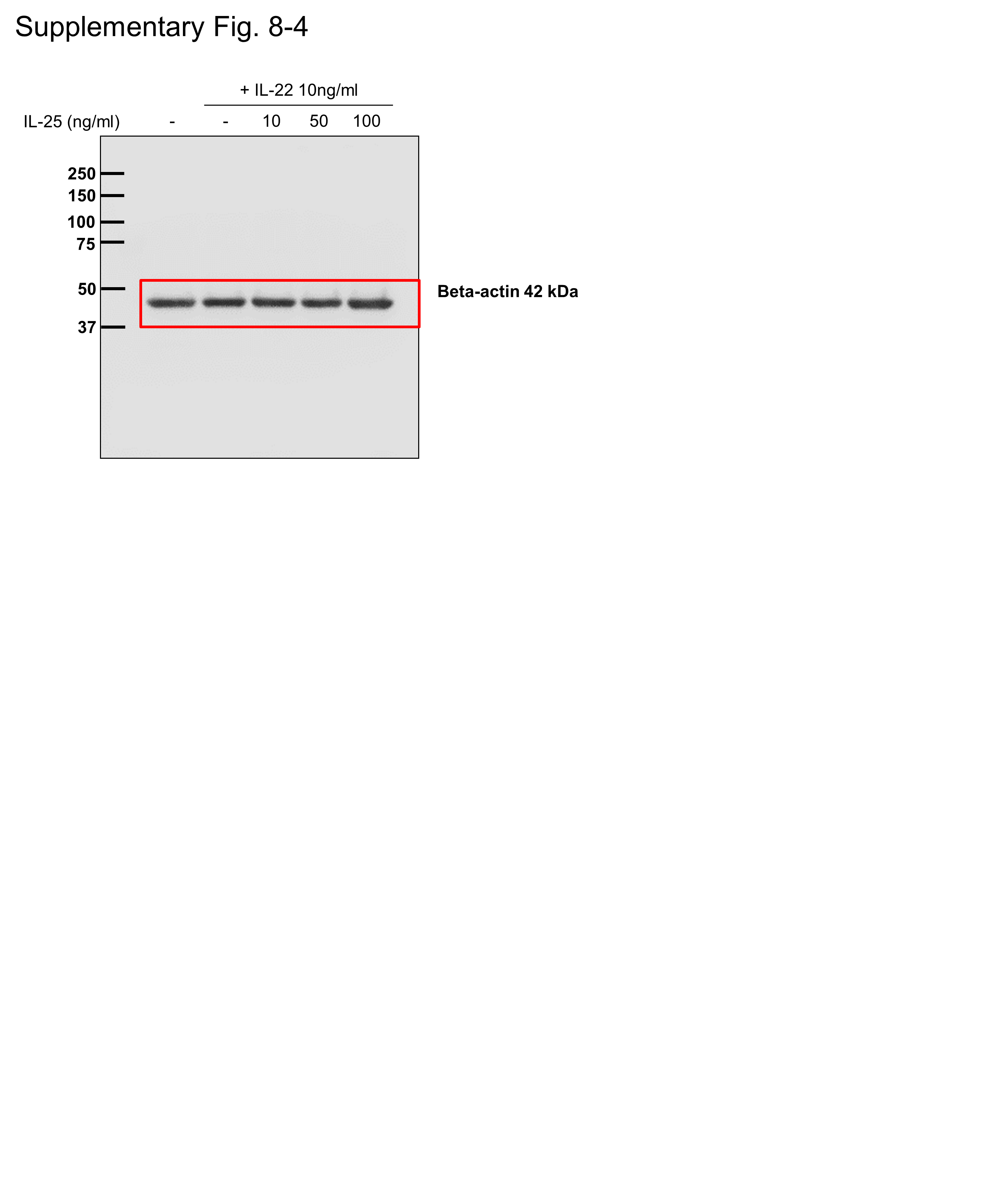

Supplement: Supplementary file 9 — Additional file 9: Supplementary Figure 8. Raw western blot data of Fig. 4 (IL-25 pre-treatment with IL-22 stimulation on PBMC). [file 13075_2020_2315_MOESM9_ESM.zip › Additional file 9-4.tif]
